# PTPN1/2 inhibition promotes muscle stem cell differentiation in Duchenne muscular dystrophy

**DOI:** 10.26508/lsa.202402831

**Published:** 2024-10-30

**Authors:** Yiyang Liu, Shulei Li, Rebecca Robertson, Jules A Granet, Isabelle Aubry, Romina L Filippelli, Michel L Tremblay, Natasha C Chang

**Affiliations:** 1 Department of Biochemistry, Faculty of Medicine and Health Sciences, McGill University , Montréal, Canada; 2 Goodman Cancer Institute, McGill University , Montréal, Canada

## Abstract

Duchenne muscular dystrophy (DMD) is a lethal disease caused by mutations in the *DMD* gene that encodes dystrophin. Dystrophin deficiency also impacts muscle stem cells (MuSCs), resulting in impaired asymmetric stem cell division and myogenic commitment. Using MuSCs from DMD patients and the DMD mouse model *mdx*, we found that PTPN1 phosphatase expression is up-regulated and STAT3 phosphorylation is concomitantly down-regulated in DMD MuSCs. To restore STAT3-mediated myogenic signaling, we examined the effect of K884, a novel PTPN1/2 inhibitor, on DMD MuSCs. Treatment with K884 enhanced STAT3 phosphorylation and promoted myogenic differentiation of DMD patient-derived MuSCs. In MuSCs from *mdx* mice, K884 treatment increased the number of asymmetric cell divisions, correlating with enhanced myogenic differentiation. Interestingly, the pro-myogenic effect of K884 is specific to human and murine DMD MuSCs and is absent from control MuSCs. Moreover, PTPN1/2 loss-of-function experiments indicate that the pro-myogenic impact of K884 is mediated mainly through PTPN1. We propose that PTPN1/2 inhibition may serve as a therapeutic strategy to restore the myogenic function of MuSCs in DMD.

## Abstract (precis)

Our study identifies PTPN1/2 as potential therapeutic targets for Duchenne muscular dystrophy and suggests the use of PTPN1/2 inhibitors to enhance the myogenic function of DMD muscle stem cells.

## Graphical Abstract

**Figure:**
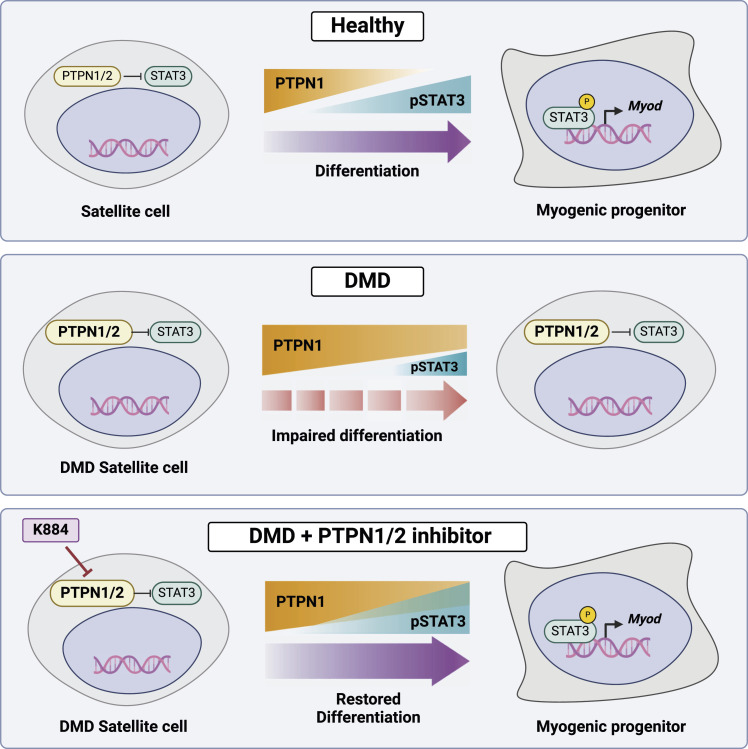

## Introduction

Duchenne muscular dystrophy (DMD) is a lethal X-linked muscle degenerative disorder characterized by progressive muscle wasting (Emery, 2002). Affecting ∼1 in every 5,000 male births worldwide, DMD represents one of the most common fatal childhood genetic diseases (Mendell & Lloyd-Puryear, 2013). DMD is caused mainly by large mutations within the *DMD* gene that lead to the absence or truncated expression of dystrophin protein (Bladen et al, 2015). Boys are diagnosed around 2–5 yr of age as signs of reduced mobility and delayed motor development become apparent (Bushby et al, 2010a). Progressive and accumulating muscle degeneration results in a loss of ambulation, and most patients are wheelchair-dependent by the age of 12 (Bushby et al, 2010b). Treatment with glucocorticoids is the current standard of care, which delays the decline of muscle function (Bushby et al, 2010a). With the help of this steroid treatment combined with cardiac and respiratory support, DMD patients can now live until 30 yr of age (Birnkrant et al, 2018). Despite intense research efforts to understand the cause and pathophysiology of DMD, there remains no effective cure.

*DMD* encodes for dystrophin, a 427 kD protein that is an integral component of the dystrophin glycoprotein complex (DGC) (Hoffman et al, 1987). The DGC, a large multi-protein complex that includes dystrophin, dystroglycans, sarcoglycans, syntrophins, dystrobrevin, caveolin, and neuronal nitric oxide synthase, spans the sarcolemma membrane of muscle cells, linking the intracellular cytoskeleton of myofibers with the surrounding extracellular matrix (Ervasti & Campbell, 1991). In the case of DMD, the absence of functional dystrophin protein disrupts the formation of the DGC, thereby weakening the sarcolemma and rendering the myofibers susceptible to contraction-induced muscle damage (Ervasti et al, 1990; Petrof et al, 1993). Moreover, altered membrane permeability in dystrophin-deficient muscle increases intracellular calcium levels, impairing mitochondrial function and activating calcium-dependent degradative proteases that contribute to muscle cell death (Turner et al, 1988; Fong et al, 1990; Millay et al, 2008).

In addition to the dystrophin-mediated loss of sarcolemma integrity in mature muscle cells, dystrophin deficiency has negative consequences on the regenerative capacity of muscle stem cells (MuSCs). MuSCs, also known as satellite cells, are muscle-resident somatic stem cells that are situated between the sarcolemma and basal lamina that encapsulate the myofiber (Mauro, 1961; Relaix & Zammit, 2012). In healthy muscles, MuSCs usually remain in a dormant, quiescent state (Schultz et al, 1978). Upon activation, often in response to damage or injury, MuSCs proliferate, giving rise to the myogenic progenitor cells required for muscle regeneration (Relaix & Zammit, 2012). MuSCs also contribute to the homeostatic maintenance of muscle tissue (Keefe et al, 2015). Hence, maintenance of the MuSC population throughout life ensures their lifelong regenerative capacity and, therefore, proper muscle health and function. Impairment of the regenerative capacity of MuSCs contributes to degenerative diseases (muscular dystrophy) and cancer (rhabdomyosarcoma) (Robertson et al, 2024).

After their activation, MuSCs can undergo a symmetric cell division, generating two daughter stem cells, or alternatively, an asymmetric cell division, which gives rise to both a daughter stem cell and a committed myogenic progenitor. This decision is mediated through the establishment of cell polarity during division (Kuang et al, 2007; Le Grand et al, 2009; Troy et al, 2012). Moreover, dystrophin in MuSCs contributes to this process by directly interacting with MAP/microtubule affinity-regulating kinase 2 (MARK2), which induces cell polarity by phosphorylating the Par-3 family cell polarity regulator (PARD3) to promote its localization at the opposite end of the cell (Dumont et al, 2015). The expression of the dystrophin complex in the basal stem cell controls the activity of p38 MAP kinase gamma (MAPK12/SAPK3) to phosphorylate and sequester coactivator-associated arginine methyltransferase 1 (CARM1/PRMT4) within the cytoplasm (Chang et al, 2018). In the apical progenitor cell, CARM1 translocates to the nucleus, where it induces transcriptional activation of myogenic factor 5 (*Myf5*), a key mediator of myogenic commitment (Kawabe et al, 2012; Chang et al, 2018). Thus, the polarized localization of these factors results in an asymmetric stem cell division, with the committed progenitor initiating the expression of genes that mediate myogenic commitment and differentiation (Kuang et al, 2007; Troy et al, 2012). These findings indicate that in MuSCs, dystrophin regulates the generation of myogenic progenitors that contribute to muscle repair.

In the context of DMD, dystrophin-deficient MuSCs exhibit impaired asymmetric cell division and reduced expression of myogenic commitment genes (Dumont et al, 2015; Chang et al, 2018). Thus, DMD MuSCs are compromised in their ability to contribute to muscle repair, thereby contributing to chronic muscle degeneration (Chang et al, 2016). Whereas promising strategies are being explored to restore dystrophin expression via gene therapy in dystrophic muscle, including CRISPR/Cas9-mediated gene editing, exon skipping, and antisense oligonucleotides, these approaches are inefficient at targeting MuSCs (Arnett et al, 2014; Verhaart & Aartsma-Rus, 2019; Filippelli & Chang, 2022). To identify novel strategies that enhance the regenerative potential of DMD MuSCs, we focused on targeting pathways that regulate the myogenic capacity of MuSCs downstream of dystrophin. Here, we examined the use of K884, a novel competitive inhibitor of protein tyrosine phosphatase non-receptor type 1 (PTPN1/PTP1B) and protein tyrosine phosphatase non-receptor type 2 (PTPN2/TC-PTP), to promote myogenic differentiation in dystrophin-deficient MuSCs (Tremblay et al, 2023).

PTPN1/2 are ubiquitously expressed, non-transmembrane phosphatases that dephosphorylate tyrosine-phosphorylated proteins (Brown-Shimer et al, 1990; Chernoff et al, 1990). They are highly homogenous, sharing 72% sequence identity within their catalytic domain, and their substrates include receptor tyrosine kinases and mediators of the Janus kinase/signal transducer and activator of transcription (JAK/STAT) signaling pathway (Pike & Tremblay, 2016). PTPN1/2 are well characterized as regulators of leptin and insulin signaling, and their dysregulations have been linked to metabolic disorders, including diabetes and obesity (Elchebly et al, 1999; Cheng et al, 2002; Zabolotny et al, 2002). PTPN1/2 are also implicated in breast and prostate cancer and exhibit both tumor-suppressive and tumor-promoting roles (Lessard et al, 2010). As such, they have become considerable targets of interest for diseases including diabetes, obesity, and cancer (Dubé & Tremblay, 2005). However, the role of PTPN1/2 in stem cell biology and the therapeutic potential of PTPN1/2 inhibitors in regenerative medicine is poorly understood. One study using MSI-1463, a naturally occurring aminosterol inhibiting PTPN1 through a non-competitive allosteric mechanism, found enhanced MuSC proliferation in mice following acute muscle injury with treatment (Smith et al, 2017). However, the mechanism and outcome of increased MuSC proliferation following MSI-1463 treatment were not explored.

Downstream of PTPN1/2, JAK/STAT signaling is involved in various cellular events, including proliferation, differentiation, and survival. In MuSCs, JAK and STAT proteins have been implicated in proliferation and differentiation. STAT3 mediates myogenic commitment by activating the basic helix-loop-helix transcription factor myogenic differentiation 1 (*Myod1*) (Tierney et al, 2014). In line with the role of STAT3 in promoting myogenic commitment, enhanced JAK2/STAT3 signaling was observed in aged MuSCs (Price et al, 2014). Increased lineage commitment during aging has been described in multiple stem cell types and contributes to age-related loss of the stem cell pool (Liu & Rando, 2011). Thus, inhibition of JAK2/STAT3 in aged MuSCs counteracted the elevated propensity towards differentiation and improved muscle regeneration in aged muscles (Price et al, 2014). In addition, genetic deletion of STAT3 specifically in MuSCs of adult mice resulted in impaired regeneration that was particularly severe in dystrophin-deficient mice (Zhu et al, 2016). Despite differences between pharmacological inhibition and genetic ablation of STAT3, these findings altogether implicate STAT3 as a key player in MuSC commitment to myogenesis and regenerative capacity.

In contrast to aging, DMD MuSCs exhibit impaired asymmetric cell division and reduced commitment to myogenesis (Dumont et al, 2015; Chang et al, 2018). We therefore hypothesized that treatment of DMD MuSCs with the PTPN1/2 inhibitor K884 would enhance their myogenic differentiation by activating JAK2/STAT3-mediated myogenic commitment and differentiation pathways. Using established and characterized human MuSC clones derived from DMD patients and unaffected controls (Massenet et al, 2020), we found that PTPN1 expression and STAT3 phosphorylation are dysregulated in DMD MuSCs. Interestingly, treatment with K884 restored the levels of STAT3 phosphorylation and enhanced myogenic differentiation specifically in DMD but not control MuSCs. Moreover, the pro-myogenic effect of K884 was dependent on PTPN1 expression and STAT3 activation. Our findings were validated in MuSCs from *mdx* mice, a mouse model of DMD (Bulfield et al, 1984). K884 treatment in *mdx* MuSCs enhanced the number of committed myogenic progenitors and increased the number of asymmetric MuSC divisions. Altogether, our results indicate that inhibiting PTPN1 through treatment with the PTPN1/2 inhibitor K884 restores activation of STAT3 and promotes myogenic differentiation of DMD MuSCs. We therefore propose that treatment of DMD MuSCs with PTPN1/2 inhibitors may serve to enhance MuSC-mediated muscle repair in dystrophic muscle.

## Results

### DMD MuSCs exhibit altered PTPN1/2 expression and STAT3 phosphorylation kinetics during myogenic differentiation

**Figure 1.**
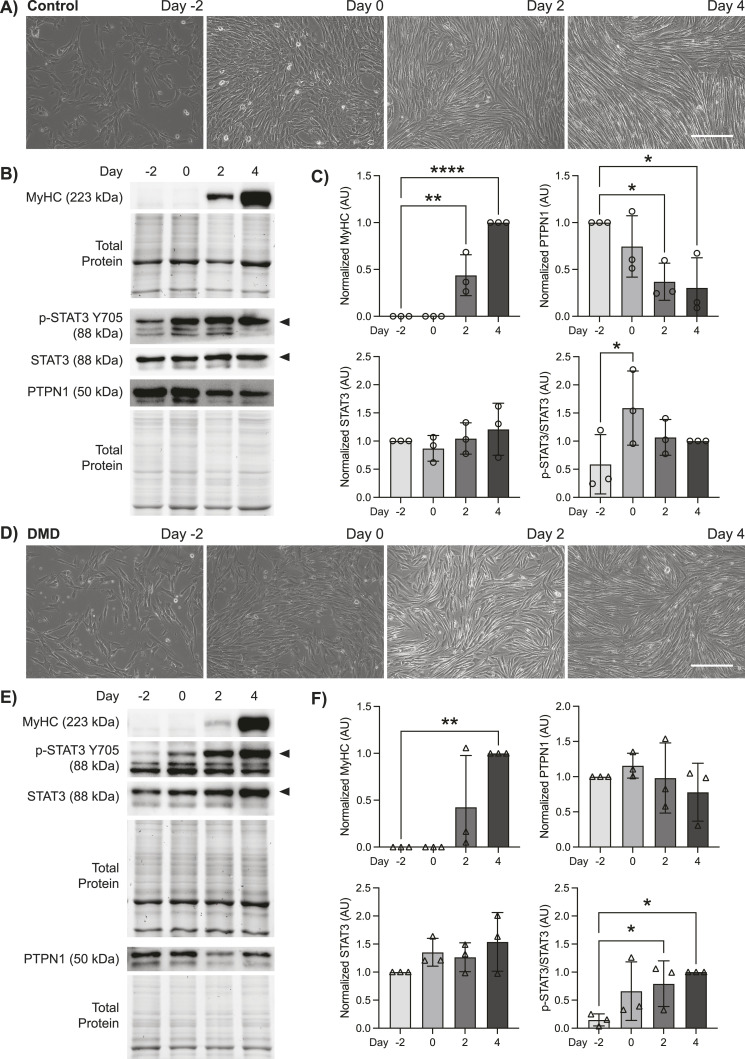
Duchenne muscular dystrophy (DMD) muscle stem cells (MuSCs) exhibit altered PTPN1 expression and STAT3 phosphorylation kinetics during myogenic differentiation. **(A)** Control human MuSCs were cultured and differentiated for 4 d. Phase contrast images were taken on days −2, 0, 2, and 4 of differentiation. Scale bar represents 150 μm. **(B)** Protein expression of MyHC, phosphorylated STAT3 (p-STAT3, Y705), total STAT3, and PTPN1 on days −2, 0, 2, 4 of differentiation in control cells were examined by immunoblot analysis (n = 3 biological replicates per group). **(C)** Quantification of MyHC, p-STAT3, STAT3, and PTPN1 protein expression levels in control cells. **(D)** DMD patient-derived MuSCs were subject to differentiation as in (A). **(E)** Immunoblot analysis and (F) quantification of indicated proteins from DMD cells as described in (B, C). MyHC, STAT3, and PTPN1 were normalized to total protein; p-STAT3 was normalized to total STAT3 (AU; arbitrary units). Data are represented as mean ± SD, *P < 0.05, **P < 0.01, ****P < 0.0001 (one-way ANOVA with Fisher’s uncorrected LSD). Source data are available for this figure.

We performed a myogenic differentiation time-course experiment to examine the expression profile of PTPN1/2 and the phosphorylation status of their substrate STAT3 in DMD patient-derived and unaffected control human MuSCs. Differentiation was initiated and assessed at 2-d intervals: 2 d before differentiation (D-2), at the beginning of differentiation (D0), as well as 2 d (D2) and 4 d (D4) post-differentiation. Both control and DMD MuSCs exhibited changes in cell morphology and fusion into myotubes by D4 post-differentiation (Fig 1A and D). Cell lysates were prepared during the differentiation time-course for immunoblot analysis. At D2 and D4 post-differentiation, myosin heavy chain (MyHC) protein expression indicated terminal differentiation of both control and DMD MuSCs to myotubes (Fig 1B, C, E, and F). Of note, in control cells MyHC was significantly increased from D-2 at D2 and D4 post-differentiation, whereas DMD cells exhibited significantly increased MyHC levels only at D4, indicating delayed myogenic differentiation in DMD cells (Fig 1C and F).

**Figure S1.**
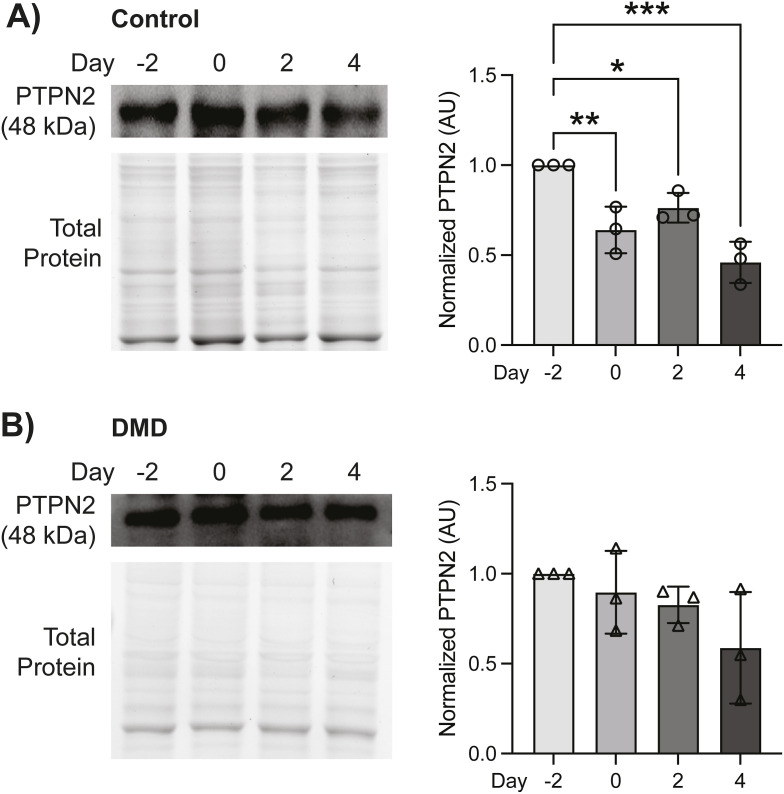
PTPN2 expression levels in control and Duchenne muscular dystrophy (DMD) muscle stem cells (MuSCs). **(A)** Control and (B) DMD human MuSCs were cultured and differentiated for 4 d as in Fig 1 (n = 3 biological replicates per group). Levels of PTPN2 protein expression during differentiation in control and DMD cells were examined by immunoblot. PTPN2 expression was normalized to total protein (arbitrary units; AU). Data are represented as mean ± SD, *P < 0.05, **P < 0.01, ***P < 0.001 (one-way ANOVA with Fisher’s LSD).

PTPN1 expression in control MuSCs was highest in undifferentiated cells, and its expression steadily decreased during differentiation, decreasing by 2.71-fold at D2 and 3.29-fold by D4 (Fig 1B and C). In contrast, protein levels of PTPN2 exhibited a more gradual decline, showing 1.32-fold reduction at D2 and 2.17-fold reduction at D4 post-differentiation (Fig S1A). These results indicate that the down-regulation of PTPN1/2, may play a role in facilitating the myogenic differentiation program. Moreover, the reduction in PTPN1/2 expression correlated with increased phosphorylation of STAT3 at tyrosine residue 705 (Y705) at the onset of differentiation (Fig 1B and C). Levels of phosphorylated STAT3 in control MuSCs were significantly increased 2.70-fold at D0 from D-2 (Fig 1C). This trend supports the notion that the down-regulation of a regulatory tyrosine phosphatase during differentiation is required to promote the phosphorylation and activation of key myogenic regulators.

In contrast to control MuSCs, PTPN1 expression levels in DMD MuSCs did not exhibit a steady reduction during differentiation, increasing slightly at D0 and decreasing by only 1.3-fold at D4 (Fig 1E and F), resulting in significant variance between control and DMD cells (*P* = 0.038). Similarly, DMD MuSCs did not exhibit as drastic a difference in PTPN2 expression levels during differentiation as compared with control cells, decreasing by 1.7-fold at D4 (Fig S1B). Of note, PTPN2 expression in control compared with DMD was not significantly different (*P* > 0.05). In accordance with these sustained PTPN1/2 levels in DMD cells, phosphorylated STAT3 levels did not peak until D4 of differentiation (Fig 1E and F). Our results suggest that PTPN1/2 may play a regulatory role in healthy MuSCs to negatively regulate STAT3 activation to maintain stemness and prevent myogenic differentiation in MuSCs. Importantly, the down-regulation of PTPN1/2 expression during differentiation and the concomitant up-regulation of STAT3 phosphorylation appear to be dysregulated in DMD MuSCs, suggesting that PTPN1/2 may be a viable target to restore pro-myogenic STAT3 signaling in DMD MuSCs.

### Treatment with the PTPN1/2 inhibitor K884 increased STAT3 phosphorylation in DMD MuSCs

**Figure 2.**
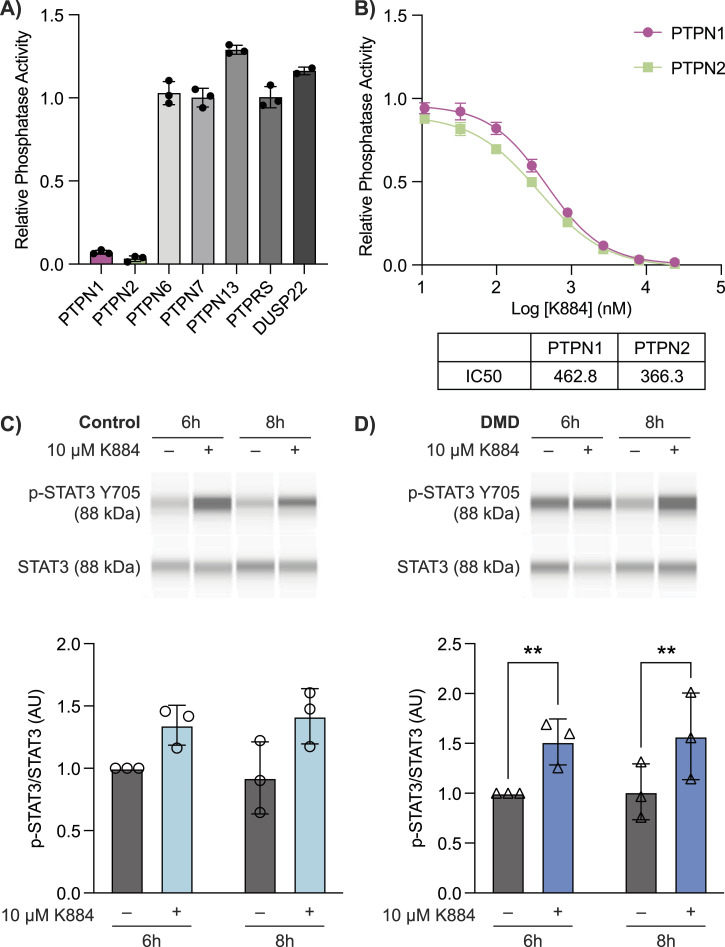
Duchenne muscular dystrophy (DMD) muscle stem cells (MuSCs) exhibit increased STAT3 phosphorylation upon treatment with the PTPN1/2 inhibitor K884. **(A)** In vitro phosphatase assays were performed with the indicated phosphatases using 20 mM DiFMUP as a substrate in the presence of 20 mM K884. Phosphatase activity (fluorescence units/minute) was determined by measuring fluorescence (excitation 358 nm, emission 455 nm) over 10 min in 30 s intervals and presented as relative phosphatase activity compared with vehicle (no inhibitor) control (n = 3 replicates). Phosphatase activity was normalized to vehicle controls. **(B)** In vitro phosphatase assays with PTPN1 and PTPN2 and DiFMUP substrate were performed in the presence of increasing concentrations of K884 from 10 nM to 24 μM. Substrate concentrations equivalent to the K_m_ value for PTPN1 (13 μM) and PTPN2 (7 μM) were used. IC50 values were derived by a sigmoidal dose-response curve using GraphPad Prism software. **(C)** Control and (D) DMD human MuSCs were treated with 10 μM K884 or vehicle (sterile water) and differentiated for 24 h (n = 3 biological replicates per group). Protein expression of p-STAT3 (Y705) and STAT3 at 6- and 8-h post-differentiation and K884 treatment were examined by Simple Western immunoassays. p-STAT3 (Y705) was normalized to total STAT3 protein levels and presented as a fold-increase compared with vehicle-treated cells at 6 h. Data are represented as mean ± SD, **P < 0.01 (two-way ANOVA with Fisher’s LSD). Source data are available for this figure.

To target PTPN1/2 in DMD MuSCs, we used a novel competitive inhibitor of PTPN1/2, K884 (Tremblay et al, 2023). Of the non-receptor, receptor-like, and dual specificity phosphatases we tested, K884 exhibited potent specificity and equivalent IC50 for both PTPN1 and PTPN2 (Fig 2A and B). To investigate the impact of PTPN1/2 inhibition on STAT3 phosphorylation during MuSC differentiation, control and DMD MuSCs were differentiated either in the presence or absence of 10 μM of K884. Cells were collected at 6- and 8-h post-treatment for quantitative capillary-based immunoassays (also known as Simple Western). In both control and DMD MuSCs, K884 treatment increased STAT3 Y705 phosphorylation when compared with vehicle-treated cells (*P* < 0.05), with 6- and 8-h post-treatment in DMD cells being significant in particular (Fig 2C and D). These results show that PTPN1/2 inhibition with K884 in MuSCs can effectively increase the levels of STAT3 phosphorylation.

### PTPN1/2 inhibition with K884 enhances myogenic differentiation of DMD MuSCs

**Figure 3.**
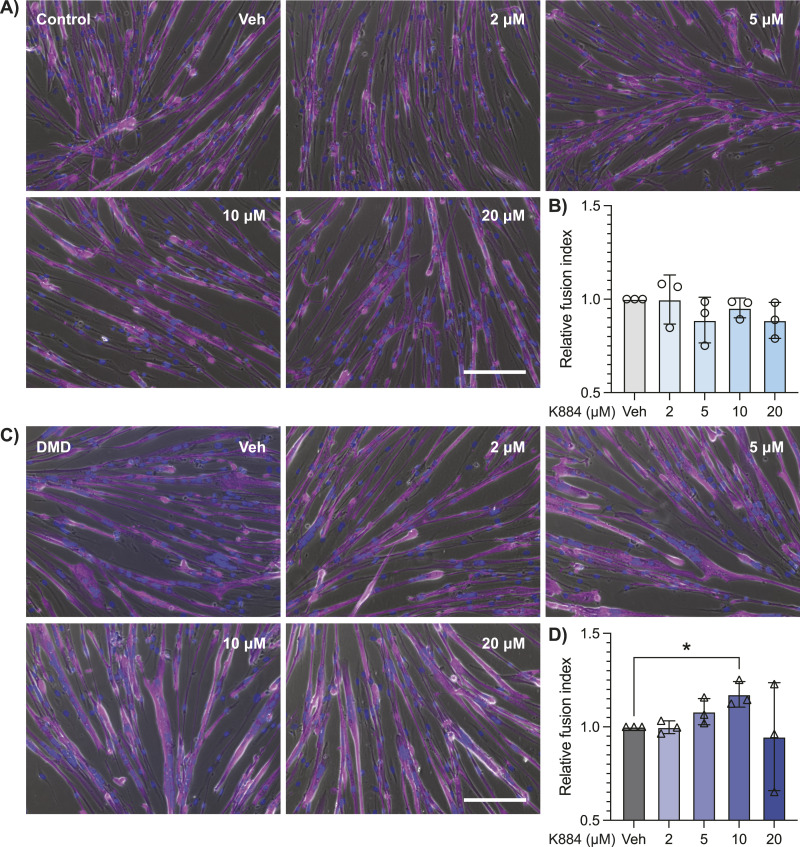
PTPN1/2 inhibition with K884 enhances myogenic differentiation of Duchenne muscular dystrophy (DMD) muscle stem cells (MuSCs). **(A)** Control human MuSCs were differentiated with 2, 5, 10, or 20 μM of K884 or vehicle for 4 d (n = 3 biological replicates per group). Cells were fixed and immunolabeled with an anti-MyHC antibody (magenta). Nuclei were counterstained with DAPI (blue). Immunofluorescence images were merged with phase contrast images. Scale bar represents 150 μm. **(B)** Differentiation efficiency of (A) was determined by quantifying the nuclear fusion index. **(C)** DMD patient-derived MuSCs were differentiated and treated as described in (A). **(D)** Nuclear fusion index of (C). Data are represented as normalized to vehicle-treated cells and as mean ± SD, *P < 0.05 (one-way ANOVA with Fisher’s LSD).

**Figure S2.**
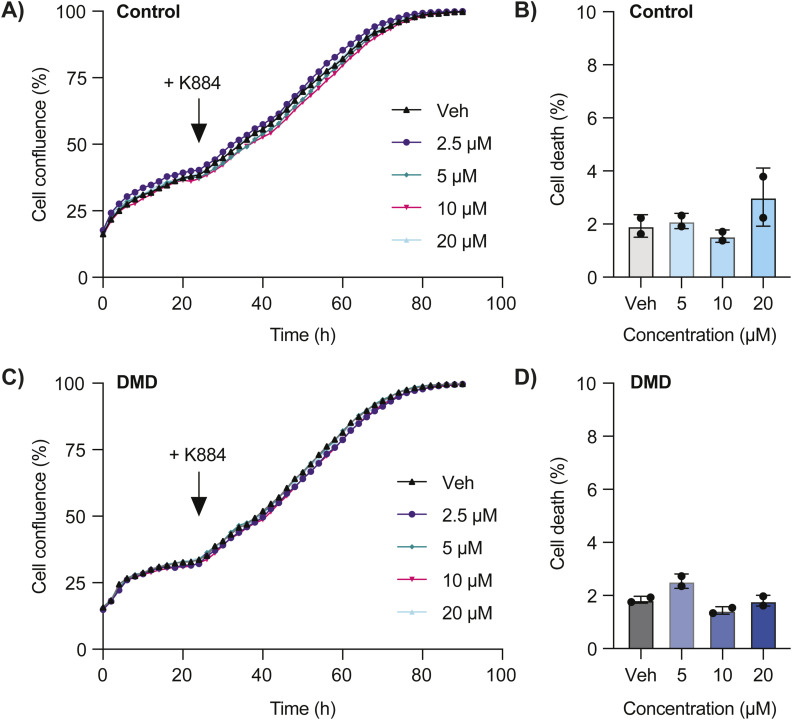
K884 does not impact cell proliferation or viability. **(A)** Cell confluency of control muscle stem cells (MuSCs) was monitored in an Incucyte cell culture system for 24 h before treatment with the indicated concentrations of K884. After treatment, cell confluency was monitored for an additional 72 h. **(B)** Control MuSCs were treated with the indicated concentrations of K884 for 48 h. Cells were stained with the viability dye eFluor 780 and the percentage of positively stained cells (% cell death) was determined by flow cytometry. **(C)** Duchenne muscular dystrophy (DMD) MuSCs were treated as described in (A). **(D)** DMD MuSCs were treated as described in (B). Data are represented as mean ± SD (n = 2 technical replicates per line). Pearson’s r = 0.999–1 for all comparisons (A, C), and *P* > 0.05, two-way ANOVA (B, D).

We hypothesized that alleviating the negative repression on STAT3 in DMD MuSCs would have a pro-myogenic effect during differentiation of these cells. To test this hypothesis, we performed in vitro differentiation assays with K884 to examine the impact of PTPN1/2 inhibition on myogenic differentiation. Control and DMD MuSCs were differentiated when treated with varying concentrations of K884 (2, 5, 10, and 20 μM). Four days post-differentiation, the differentiation efficiency was quantified by assessing nuclear fusion index within multinucleated myotubes (Fig 3A and C). Treatment with 2–20 μM K884 had no significant impact on the differentiation of control MuSCs (Fig 3A and B). In contrast, treatment of DMD MuSCs with 10 μM K884 resulted in a significant increase in fusion index (*P* = 0.047, Fig 3C and D). Importantly, cell proliferation (Fig S2A and C) and viability (Fig S2B and D) are not impacted at these concentrations of K884 in control and DMD MuSCs. Altogether, these results indicate that DMD MuSCs are more responsive to PTPN1/2 inhibition and exhibit enhanced myogenic differentiation after treatment with 10 μM of K884.

### Depeletion of PTPN1 in DMD MuSCs abrogates the pro-myogenic effect of K884

**Figure 4.**
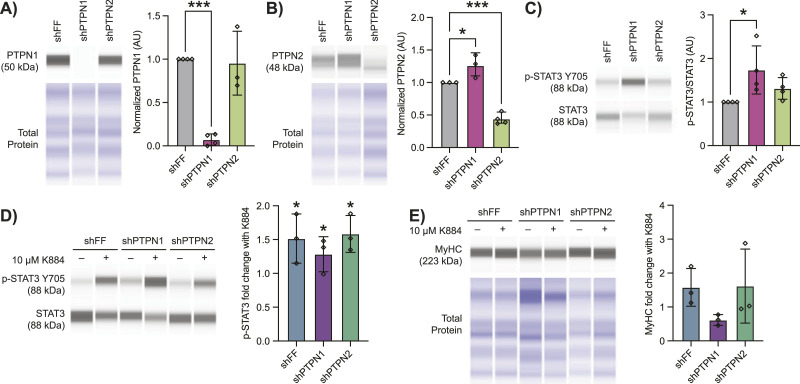
The pro-myogenic effect of K884 in Duchenne muscular dystrophy (DMD) muscle stem cells (MuSCs) is mediated mainly through PTPN1. DMD muscle stem cells were treated with either control (shFF) or shRNA targeting PTPN1 (shPTPN1) or PTPN2 (shPTPN2). **(A, B, C)** Simple Western immunoassays of cell lysates from shRNA-treated cells examining (A) PTPN1, (B) PTPN2, and their (C) STAT3 and p-STAT3 levels (n = 3 or 4 replicates, as shown). **(D, E)** After shRNA treatment, cells were differentiated either in the presence or absence of 10 μM K884 (sterile H_2_O was used as vehicle). Cells were collected (D) 6 h after initiating differentiation and protein levels of STAT3 and p-STAT3 were quantified (n = 3 replicates), or (E) 4 d after differentiation and protein levels of MyHC were quantified (n = 3 replicates) by Simple Western. **(C, D)** In (A, B, E), MyHC, PTPN1, and PTPN2 protein levels were normalized to total protein, and in (C, D), p-STAT3 was normalized to total STAT3 (AU; arbitrary units). Data represented in (A, B, C) are normalized to shFF. Data represented in (D, E) are represented as fold-change with K884. Data are represented as mean ± SD, *P < 0.05, ***P < 0.001 (two-way ANOVA with Fisher’s LSD). Source data are available for this figure.

Based on our findings that PTPN1 is more differentially expressed during differentiation of control MuSCs and shows increased dysregulation in DMD as compared with PTPN2, we predicted that the pro-myogenic effect of K884 is mediated through inhibition of PTPN1. To resolve which phosphatase has the greater role in regulating the phosphorylation status of STAT3 in human DMD MuSCs, we used short-hairpin RNA (shRNA) to knockdown both PTPN1 (shPTPN1) and PTPN2 (shPTPN2), and a control firefly luciferase shRNA (shFF) (Fig 4A and B). We found that inhibition of either PTPN1 or PTPN2 resulted in increased levels of phosphorylated STAT3; however, depletion of PTPN1 resulted in a larger and significant increase in phosphorylation of STAT3 compared with shPTPN2 (1.74- and 1.31-fold, respectively) (Fig 4C). To address the importance of either PTPN1 or PTPN2 expression in mediating the pro-myogenic effect of K884, we differentiated shFF, shPTPN1 and shPTPN2 human DMD MuSCs with vehicle or K884. At 6 h post-treatment, differentiation with K884 treatment resulted in an increase in phosphorylated STAT3 in all conditions, with a fold-increase of 1.52 in shFF, 1.28 in shPTPN1, and 1.58 in shPTPN2 (Fig 4D). Moreover, when we examined the cells following 4 d of differentiation, we found that K884 treatment increased the expression of MyHC in shFF and shPTPN2 cells (1.58-fold and 1.62-fold, respectively), but this effect of K884 was abrogated in shPTPN1 cells (0.62-fold) (Fig 4E). These data suggest that the pro-myogenic effect of K884 is mainly acting through the inhibition of PTPN1 rather than PTPN2.

### Inhibition of STAT3 activation abrogates the pro-myogenic effect of K884 in DMD MuSCs

**Figure 5.**
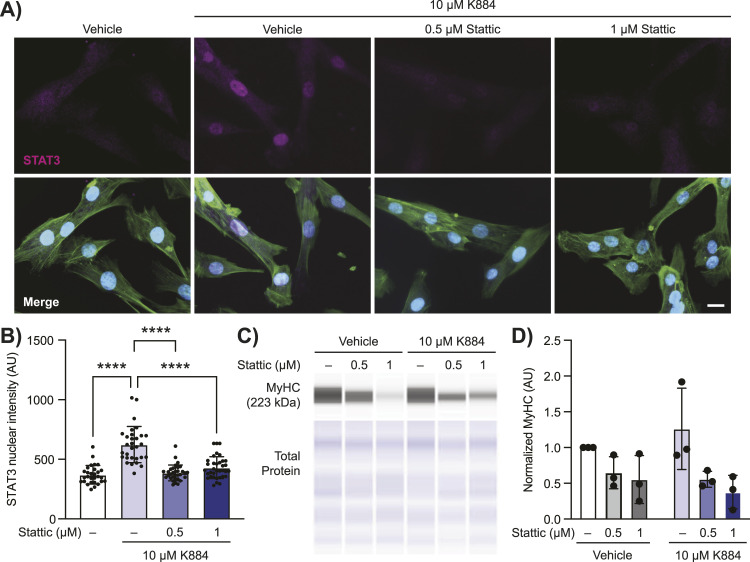
Inhibition of STAT3 activity prevents myogenic differentiation and is not rescued by K884 in Duchenne muscular dystrophy (DMD) muscle stem cells (MuSCs). **(A, B)** Duchenne muscular dystrophy muscle stem cells were treated with either DMSO (vehicle), 0.5 or 1 μM of the STAT3 inhibitor Stattic with 10 μM K884 and immunolabelled for STAT3 (magenta), phalloidin (green), and Hoechst (blue), and (B) nuclear STAT3 intensity was quantified (n = 29, 35, 32, and 35 cells, respectively). Scale bar represents 20 μm. **(C, D)** Cell lysates for cells treated as in (A) were assessed for MyHC levels by Simple Western (n = 3 replicates). Levels of p-STAT3 were normalized to total STAT3 (AU; arbitrary units). Data are represented as normalized to vehicle-treated cells. Data are represented as mean ± SD, ****P < 0.0001 (one-way ANOVA (B) and two-way ANOVA (D) with Fisher’s LSD). Source data are available for this figure.

Phosphorylation of STAT3 at Y705 induces STAT3 dimerization and translocation to the nucleus where it is transcriptionally active and this phosphorylation event has been observed in activated MuSCs undergoing regeneration (Zhong et al, 1994; Tierney et al, 2014). We asked if the enhanced myogenic differentiation potential of K884 is because of its ability to neutralize PTPN1/2-mediated inhibition of STAT3 activation. To test this, we performed differentiation assays in the presence of the STAT3 inhibitor Stattic. Stattic is a non-peptidic small molecule that selectively binds the SH2 domain of STAT3, thereby preventing STAT3 dimerization (independent of STAT3 phosphorylation status) and thus STAT3 transcriptional activity (Schust et al, 2006). To validate the effect of K884 and Stattic on STAT3 nuclear translocation, we differentiated DMD MuSCs in the presence of vehicle, 0.5 or 1 μM of Stattic with K884, and performed immunofluorescence assays for STAT3 (Fig 5A). As expected, K884 treatment induced STAT3 nuclear translocation (Fig 5A and B). Moreover, treatment with 0.5 and 1 μM Stattic prevented the ability of K884 to induce STAT3 nuclear translocation (Fig 5A and B). We then assessed the differentiation capacity of DMD MuSCs in the presence of K884 and Stattic. Treatment of DMD MuSCs with Stattic alone or in conjunction with K884 had a negative impact on myogenic differentiation as assessed by MyHC levels (*P* = 0.038), which was not rescued by the addition of K884 (*P* = 0.99) (Fig 5C and D). These results indicate the importance of STAT3 transcriptional activity in differentiation and the inability of K884 treatment to overcome its inhibition. Given this and the ablation of K884-mediated STAT3 nuclear translocation by Stattic, these results support the conclusion that the pro-myogenic effect of K884 is mediated through its ability to activate STAT3.

### K884 treatment promotes asymmetric MuSC divisions in a mouse model of DMD

**Figure 6.**
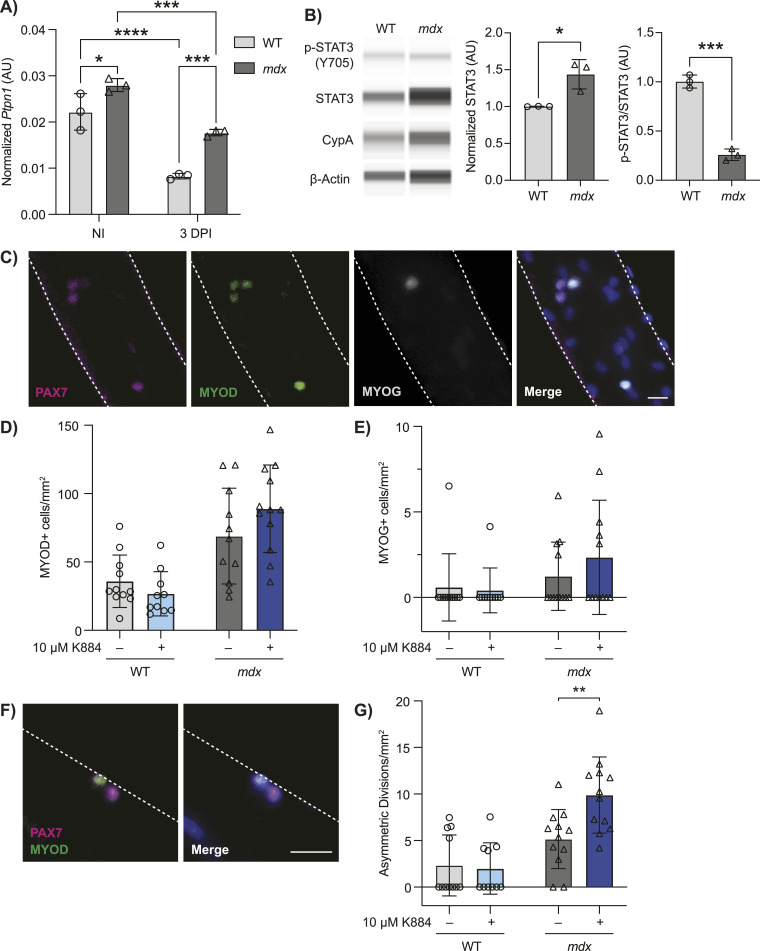
K884 treatment promotes asymmetric muscle stem cell (MuSC) divisions in a mouse model of Duchenne muscular dystrophy. **(A)** MuSCs were prospectively isolated from non-injured (NI) and 3 d post-injured (3 DPI) hind limb muscles from WT and *mdx* mice (n = 3 biological replicates). Expression of *Ptpn1* was determined by droplet digital PCR analysis and normalized to the geomean of *Rps18* and *Rps20* reference genes (AU; arbitrary units). **(B)** Quantification of STAT3 and p-STAT3 protein levels by Simple Western from isolated MuSCs from WT and *mdx* mice (n = 3 biological replicates). STAT3 levels were normalized to the geomean of cyclophilin A (CypA) and β-actin protein expression to account for loading. p-STAT3 was normalized to total STAT3 (AU; arbitrary units). Both STAT3 and p-STAT3/STAT3 quantifications are represented as relative to WT control. **(C)** Single myofibers were isolated from extensor digitorum longus muscles of WT and *mdx* mice. The representative images of myofibers immunolabeled with antibodies against PAX7 (magenta), MYOD (green), and MYOG (grey) are shown. Nuclei were counterstained with Hoechst (blue). Scale bar represents 20 μm. **(D, E)** Numbers of (D) MYOD+ and (E) MYOG+ cells from vehicle or 10 μM K884 treated WT and *mdx* myofibers normalized to myofiber area. **(F)** Representative image of a MuSC asymmetric cell division; Pax7^high^MyoD^low^ and Pax7^low^MyoD^high^ cell doublet. Scale bar represents 20 μm. **(G)** Numbers of asymmetric MuSC divisions from vehicle or 10 μM K884 treated WT and *mdx* myofibers normalized to myofiber area. Data are represented as mean ± SD, *P < 0.05, **P < 0.01, ***P < 0.001, ****P < 0.0001 (two-way ANOVA with Fisher’s LSD (A) and two-sided unpaired *t* test). Source data are available for this figure.

**Figure S3.**
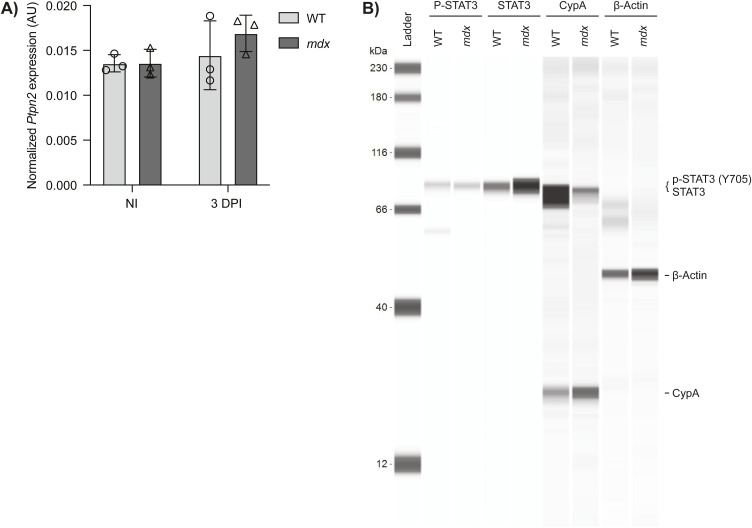
*Ptpn2* expression and quantification of p-STAT3 in murine muscle stem cells (MuSCs). **(A)** MuSCs were prospectively isolated from non-injured (NI) and 3 d post-injured (3 DPI) hind limb muscles from WT and *mdx* mice (n = 3 biological replicates, n.s. by unpaired two-tailed *t* test). Expression of *Ptpn2* was determined by droplet digital PCR analysis and normalized to the geomean of *Rps18* and *Rps20* reference genes (AU; arbitrary units). **(B)** Pseudo-blot of Fig 5B showing full lane view of p-STAT3, STAT3, cyclophilin A (CypA), and β-actin protein levels by Simple Western from isolated MuSCs from WT and *mdx* mice.

To validate our findings in human DMD MuSCs, we used the *mdx* mouse model of DMD, which harbors a spontaneous mutation in *Dmd*, resulting in a premature stop codon within exon 23, thereby ablating dystrophin expression (Bulfield et al, 1984). The WT counterparts of *mdx* mice, C57BL/10ScSn, were used as controls. We first determined the expression of *Ptpn1* and *Ptpn2* by droplet digital PCR in prospectively isolated MuSCs from the hindlimb muscles of WT and *mdx* mice. To determine if *Ptpn1/2* expression is regulated during myogenic differentiation in vivo, we collected MuSCs from resting muscle (non-injured, homeostatic MuSCs) and compared them with MuSCs isolated from regenerating muscle (3-d post-injury, activated MuSCs). Similar to PTPN1 protein levels in human MuSCs, *Ptpn1* expression is significantly down-regulated during MuSC activation in WT mice (2.68-fold decrease) (Fig 6A). Consistent with our findings in DMD MuSCs, *Ptpn1* reduction during activation was significantly less in *mdx* MuSCs (1.58-fold decrease, *P* < 0.0001) (Fig 6A). In addition, dystrophin-deficient MuSCs from *mdx* mice exhibited significantly higher levels of *Ptpn1* in both homeostatic (non-injured) and activated MuSCs (3 d post-injury) compared with WT (1.26-fold and 2.14-fold, respectively) (Fig 6A). In contrast to human MuSCs, *Ptpn2* expression in mice does not appear to be differentially regulated during MuSC differentiation or impacted by dystrophin deficiency (Fig S3A).

Using quantitative capillary-based immunoassays, we also determined the levels of STAT3 protein and STAT3 phosphorylation in prospectively isolated homeostatic *mdx* and WT MuSCs. Whereas *mdx* MuSCs exhibited increased levels of total STAT3 protein compared with WT (1.43-fold increase), the ratio of phosphorylated STAT3 (Y705) to total STAT3 protein was 3.86-fold lower in *mdx* compared with WT MuSCs (Figs 6B and S3B), much like the decrease observed in human MuSCs. Altogether, our results indicate impaired regulation of PTPN1 expression and reduced levels of STAT3 phosphorylation in human and murine DMD MuSCs compared with unaffected controls during myogenic differentiation.

To confirm the pro-myogenic effect of K884 in murine MuSCs, we used an ex vivo model of MuSC activation and differentiation. Single myofibers were isolated from the extensor digitorum longus (EDL) muscles of WT and *mdx* mice. EDL myofibers were treated with 10 μM K884 or water (vehicle control) for 48 h in a culture medium that promotes MuSC activation and proliferation. Myofibers were fixed and immunolabeled for the MuSC marker PAX7 and myogenic commitment and differentiation markers MYOD and MYOG (Fig 6). We observed a trend towards increased MYOD+ (1.29-fold) and MYOG+ (1.89-fold) *mdx* MuSCs with K884 treatment, which was not seen in WT MuSCs (Fig 6D and E).

Reduced asymmetric cell divisions in *mdx* mice are a defining characteristic of MuSC dysfunction in DMD (Dumont et al, 2015). By culturing MuSCs ex vivo on isolated single EDL myofibers, we could visualize MuSCs shortly after cell division within the first 48 h after isolation and treatment with K884. We quantified the number of asymmetric MuSC divisions, defined as MuSC doublets containing one PAX7^high^/MYOD^low^ cell and one PAX7^low^/MYOD^high^ cell (Zammit et al, 2004) (Fig 6F). Treatment with K884 significantly increased the number of asymmetric MuSC cell divisions in *mdx* myofibers (1.92-fold) and had a negligible impact on asymmetric cell divisions in WT myofibers (Fig 6G). These data support our findings from human DMD MuSCs and demonstrate that K884 exerts a pro-myogenic effect specifically in dystrophin-deficient MuSCs.

## Discussion

The JAK/STAT-signaling axis plays an integral role in communicating extracellular signals from cytokines and growth factors to influence the nuclear transcription of factors involved in cell proliferation, differentiation, and survival. Thus, tight regulation of JAK/STAT signaling is critical to prevent aberrant cell signaling associated with diseases including cancer, autoimmunity, neurodegenerative disease, and sarcopenia (O'Shea et al, 2015). Our results identify PTPN1 as a potential regulator of stemness in MuSCs, which acts to prevent STAT3 activation and differentiation. Both PTPN1 gene and protein expression is down-regulated after the initiation of differentiation in control MuSCs (Figs 1B and 6A), and this down-regulation is correlated with increased phosphorylation of STAT3. Interestingly, PTPN1 expression in DMD MuSCs did not exhibit the same downward trend during differentiation, resulting in delayed and reduced phosphorylation of STAT3, thereby suggesting impaired PTPN1/STAT3 signaling in DMD MuSCs.

Based on the premise that PTPN1 and STAT3 signaling are dysregulated in DMD MuSCs, we used K884, a novel PTPN1/2 inhibitor, to alleviate the negative repression on STAT3 activation in DMD MuSCs. Using two independent functional differentiation assays in two different DMD experimental models, we observed enhanced myogenesis after K884 treatment in human DMD and murine *mdx* MuSCs (Figs 3C and 6C–G), but not in their respective controls (Figs 3A and 6C–G). These results suggest a unique impairment in dystrophin-deficient MuSCs that is restored through treatment with K884.

PTPN1/2 are highly homologous and there are currently no competitive inhibitors that are selective for one or the other enzyme (Perez-Quintero et al, 2024). Because K884 inhibits both PTPN1 and PTPN2, we used shRNA to deplete either phosphatase in human DMD MuSCs to determine if the pro-myogenic effect of K884 was mediated by either one or both phosphatases (Fig 4A and B). Even in the absence of K884 treatment, we found that depletion of PTPN1 resulted in a greater increase in the levels of STAT3 phosphorylation compared with control cells than that of PTPN2 (Fig 4C). This result suggests that in DMD MuSCs, PTPN1 is mainly responsible for negatively regulating STAT3 transcriptional activity by maintaining it in an unphosphorylated state. Knockdown of PTPN1 alleviates this negative repression, thereby resulting in enhanced phosphorylation of STAT3. Importantly, the knockdown of PTPN1 also diminished the pro-myogenic effect of K884 (Fig 4E). Altogether these results strongly indicate that the pro-myogenic effect of K884 is mainly through inhibition of PTPN1 rather than PTPN2 and that PTPN1 itself contributes to the negative regulation of STAT3 activation in MuSCs. Whereas our results do not indicate any negative impact of PTPN2 inhibition on myogenesis, we cannot exclude the contribution of PTPN2 in K884 treatment and the impact of inhibiting both phosphatases will need to be investigated in the context of developing PTPN1/2 inhibitors for therapeutic application in DMD.

Our observations with K884 enhancement of asymmetric MuSC divisions are consistent with a previous study that reported a rise in asymmetric cell divisions after EGFR stimulation in *mdx* MuSCs (Wang et al, 2019). Activation of EGFR, which is also a target of PTPN1, in DMD MuSCs rescued cell polarity, restored asymmetric cell divisions, and improved regeneration (Flint et al, 1997; Wang et al, 2019). STAT3 is an established downstream target of EGFR, thus implicating STAT3 as a critical mediator of the pro-myogenic impact observed here and in the study by Wang et al (2019). Supporting this hypothesis, a study using the FDA-approved drug Sunitinib, a multitargeted receptor tyrosine kinase inhibitor, to treat *mdx* mice found that Sunitinib stimulated STAT3 activation and promoted muscle regeneration (Fontelonga et al, 2019). Indeed, we found that inhibiting the activation of STAT3 with the small molecule Stattic prevented the ability of K884 to induce STAT3 activation and blunted its pro-myogenic effect (Fig 5). Whereas these studies support the therapeutic benefit of STAT3 activation, the impact of transient versus sustained STAT3 activation on muscle regeneration warrants examination. Balancing stem cell self-renewal and commitment is critical for maintenance of stem cell populations (Chang et al, 2016). As STAT3 activation drives asymmetric divisions, further studies should address how K884 treatment impacts the long-term maintenance of the MuSC population (Price et al, 2014).

Whereas, in this study, we focused on STAT3, a well characterized substrate of PTPN1/2 and established mediator of myogenesis, it is likely that K884 impacts the phosphorylation of other PTPN1/2 substrates that also exerts influence on myogenic differentiation. Additional PTPN1/2 substrates include EGFR and the insulin-like growth factor 1 receptor (IGF1R), which have established roles in MuSC differentiation and myotube formation (Buckley et al, 2002; Wang et al, 2019). Moreover, as STAT3 phosphorylation is downstream of EGFR and IGF1R, the impact of K884 on STAT3 phosphorylation may be indirectly mediated through activation of these receptor tyrosine kinases.

In conclusion, our studies suggest that PTPN1/2 may be a potential target for DMD. From a therapeutic perspective, PTPN1/2 represent exciting targets because of their association with numerous diseases, including diabetes, obesity, cancer, and autoimmunity. Our findings provide compelling evidence to support further investigation of PTPN1/2 inhibition in pre-clinical contexts, including DMD mouse models. As PTPN1/2 negatively regulates inflammation and PTPN1/2 inhibition is currently being explored for immunotherapy against cancer, it would be pertinent to assess the impact of PTPN1/2 inhibition on inflammation within the context of DMD and steroid treatment (Manguso et al, 2017; Baumgartner et al, 2023).

Whereas PTPN1/2 inhibition does not restore dystrophin expression, this strategy directly addresses the impaired regenerative capacity of DMD MuSCs. Over 7,000 mutations have been reported in DMD (Bladen et al, 2015). Thus, restoring MuSC function would be predicted to benefit all DMD patients, regardless of the nature of the *DMD* mutation. We propose that a combinatorial strategy that targets MuSCs to enhance their differentiation potential combined with gene correction strategies that restore dystrophin expression in muscle tissue should be explored. We predict that enhancing endogenous repair would synergize treatments to improve muscle function and thus contribute to long-term muscle health and regenerative capacity.

## Materials and Methods

### Human MuSC culture and differentiation

Immortalized human MuSCs from unaffected individuals (Control 1–clone D52, and Control 3–clones A42 and A11) and DMD patients (DMD 2–clone G82, DMD 4–clone B42, and DMD 5–clone E82) were kindly provided by Dr. Bénédicte Chazaud (Institut NeuroMyoGène) (Massenet et al, 2020). Ethics approval was obtained from the Faculty of Medicine and Health Sciences Institutional Review Board at McGill University (A03-M23-21B).

Control and DMD MuSCs were cultured in Skeletal Muscle Cell Growth Medium (PromoCell) with Growth Medium SupplementMix (PromoCell), 10% FBS (Gibco), 40 ng/ml gentamicin (Gibco), and 1.5X GlutaMAX (Gibco). All human MuSCs were cultured at 37°C and 5% CO_2_. To passage the cells, cells were grown to 65–75% confluency, washed with 1X PBS, and trypsinized at 37°C using 1X TrypLE Express (Gibco). For differentiation assays, cells were grown to 80–85% confluency. Cells were differentiated in Skeletal Muscle Differentiation Medium (PromoCell) with Differentiation Medium SupplementMix (PromoCell), and 1% penicillin/streptomycin. Cells were differentiated at 37°C and 5% CO_2_ for up to 4 d.

### Animals and muscle regeneration

Animal work was approved by the Animal Compliance Office of McGill University, Canada (MCGL-8124). Six to nine-week-old male C57BL/10ScSnJ (B10) and C57BL/10Scsn-*Dmd*^*mdx*^/J (*mdx*) mice were used for MuSC and single myofiber isolation experiments. To induce muscle regeneration, mice were subjected to intramuscular injection of 30 μl 10 μM cardiotoxin (Latoxen Laboratory) into the tibialis anterior muscle. Mice were euthanized by cervical dislocation after anesthesia via isoflurane.

### MuSC isolation

MuSCs were isolated from B10 or *mdx* mice by fluorescence-activated cell sorting (FACS) (Pasut et al, 2012). Hindlimb muscles were dissected, minced, and dissociated with collagenase B (Roche) and dispase II (MilliporeSigma) solution using the gentleMACS Octo Dissociator with Heaters (Miltenyi Biotec). Muscle lysates were further homogenized using a syringe with an 18G X 1½ needle, then filtered through a 100 μm nylon filter, and cells pelleted by centrifugation. Cell pellets were resuspended in red blood cell lysis buffer (MilliporeSigma) and washed with FACS buffer (5% FBS, 1 mM EDTA in PBS). Cells were incubated with indicated antibodies. MuSCs were sorted based on negative lineage markers (CD11b^−^, SCA1^−^, CD45^−^, CD31^−^) and positive selection markers α7-integrin and VCAM1. Cell sorting was performed using a BD FACSAria III (BD Biosciences).

### Single myofiber isolation and culture

Single myofibers were isolated from the EDL muscle of B10 and *mdx* mice (Pasut et al, 2013). The EDL was digested in a collagenase solution 3 mg/ml collagenase type I (Worthington Biologicals) in DMEM (Gibco) and 1% penicillin/streptomycin and incubated at 37°C and 5% CO_2_ for 1.5 h. After incubation, fibers were physically separated via trituration and transferred to myofiber media (20% FBS, 1% chick embryo extract, 1% penicillin/streptomycin and 0.25% bFGF in DMEM) containing either vehicle (sterile milliQ H_2_O) or 10 μM K884 and cultured for 24 h, after which point media was replaced with untreated myofiber media and cultured for an additional 24 h (48 h total).

### Treatment with K884 and Stattic compounds

K884 was a kind gift from Kanyr Pharma Inc. K884 was dissolved in either differentiation or myofiber media at the indicated concentrations (Tremblay et al, 2023). Sterile milliQ H_2_O was used as vehicle control. Stattic (Cayman Chemicals) was dissolved in DMSO and used at a final concentration of 0.5 or 1 μM in differentiation media.

### Plasmids, lentiviral production, and infection

For knockdown of PTPN1 and PTPN2 with lentiviral shRNA, target sequences were cloned into the pPRIME-CMV-GFP-Mir-GFK-Puro plasmid backbone, which was a gift from Stephen Elledge (RRID:Addgene_11663) (Stegmeier et al, 2005). The control vector, shFF, contains a sequence targeting firefly luciferase hairpin. Individual shRNA target sequences are described in Table S1. Lentivirus production was performed as previously described, where 293T cells were transfected with individual lentiviral plasmids, the packaging (psPAX2) and envelope (pMD2.G) plasmids using CaCl_2_ (Vinette et al, 2021). Virus-containing media were collected 24- and 36-h post-transfection. Human MuSCs were infected for 8 h and selected by FACS for GFP.

Table S1. Sequences for individual shRNAs.

### In vitro phosphatase assay

In vitro phosphatase assays to determine the specificity of K884 to PTPN1 and PTPN2 and the IC50 assays were carried out as previously described (Perez-Quintero et al, 2023 *Preprint*). In brief, reactions were conducted in assay buffer (50 mM HEPES pH 7.0, 3 mM DTT, 1 mg/ml BSA) using DiFMUP (Invitrogen) as substrate. For the phosphatase screen, GST-tagged catalytic domain or full-length phosphatases were used: GST-PTPN1 (aa 1–321), GST-PTPN2 (aa 1–354), GST-PTPN6 (aa 243–595), GST-PTPN7 (aa 21–361), GST-PTPN13 (aa 2,169–2,486), GST-PTPRS (aa 883–1,501 from BC104812), GST-DUSP22 (aa 1–184). Phosphatases were pre-incubated with 20 μM K884 for 2 min before the addition of 20 μM DiFMUP substrate. Hydrolysis of DiFMUP was monitored by measuring fluorescence (excitation 358 nm/emission 455 nm) over 10 min in intervals of 30 s with a Spectramax i3 plate reader (Molecular Devices). For IC50 assays, serial dilutions of K884 (24 mm–10 nM) were added to PTPN1 and PTPN2 in assay buffer. DiFMUP substrate concentration equivalent to the K_m_ value for PTPN1 (13 μM) and PTPN2 (7 μM) were used. The dose-response curve and IC50 were determined using GraphPad Prism software (version 10.0.2).

### Cell proliferation and viability assays

To assess the impact of K884 on cell proliferation, human MuSCs were cultured in an Incucyte S3 cell culture system (Essen Bioscience). Phase contrast images of live cells were captured every 2 h for 96 h (two images per well with a 10X objective). Cell confluence was determined by creating a mask of phase contrast images using the basic analyzer module in the Incucyte S3 software (v2019A). To assess the impact of K884 on cell viability, human MuSCs were treated with K884 for 48 h. Cells were collected and stained with eFluor 780 (1:2,000; eBioscience). Flow cytometry data acquisition from stained cells was obtained with the BD LSRFortessa (BD Biosciences) and analyzed using FlowJo software (v10.10.0).

### Immunofluorescence (IF) microscopy

Human MuSCs were fixed with 4% PFA and permeabilized with 0.1% Triton X-100 and 0.1 M glycine in 1X PBS at RT. Cells were blocked with a blocking solution (5% donkey serum, 2% BSA, 0.1% Triton X-100, and 0.05% Tween-20 in 1X PBS) for 1 h at RT followed by incubating with primary antibody diluted in blocking solution overnight at 4°C. Secondary antibody incubation was performed in a blocking solution for 1 h at RT, then mounted with Prolong Gold Antifade Reagent with DAPI (Invitrogen).

Myofibers were fixed with 2% PFA and permeabilized with 0.1% Triton X-100 and 0.1 M glycine in 1X PBS at RT. Myofibers were treated with blocking solution (5% donkey serum, 2% BSA, 0.1% Triton X-100 in PBS) for 2 h at RT, then incubated with primary antibody diluted in myofiber IF solution (0.5% donkey serum, 0.5% goat serum in PBS) overnight at 4°C. Secondary antibody incubation was performed in myofiber IF solution at RT for 1 h, then stained with Hoechst (Life Technologies) and mounted with Prolong Gold Antifade Reagent (Invitrogen). Fluorescent and phase contrast images were captured using the EVOS M5000 inverted fluorescence microscope (Thermo Fisher Scientific) with a 20X objective (0.45 numerical aperture) using the EVOS M5000 software (v1.6.1899.478).

### Image analysis and determination of nuclear fusion index

To determine the nuclear fusion index of differentiated MuSCs, ImageJ Fiji software (v1.54f) was used (Schindelin et al, 2012). Single nuclei that are unfused and fused within MyHC-stained and multinucleated myotubes (containing two or more nuclei) were manually labeled and enumerated, counting at least 300 nuclei per condition (VanGenderen et al, 2022). The fusion index was calculated using the following equation:

$$Fusion\;index\;\left(\%\right)=\frac{Number\;of\;fused\;nuclei}{Total\;number\;of\;nuclei}\times 100$$

Nuclear intensity of STAT3 was determined with ImageJ Fiji software by calculating the mean grey value of the nuclear area, which was determined by creating a mask using the Hoechst channel. For EDL myofiber analysis, whole myofibers were captured at 4X magnification on an EVOS M5000 microscope and the area for each myofiber was determined by tracing the myofiber perimeter using the custom shape tool with the EVOS software (v1.6.1899.478).

### Preparation of protein lysate and immunoblot analysis

Cell pellets were collected and lysed in lysis buffer (50 mM Tris pH 7.5, 150 mM NaCl, 2 mM MgCl_2_, 0.5 mM EDTA, 0.5% Triton X-100, 1X protease inhibitor, and 1X phosphatase inhibitor) on ice for 30 min, followed by centrifugation at 15,000 rcf at 4°C for 20 min and supernatant collected. The protein concentration of cell lysates was determined using the Pierce BCA Assay Kit (Thermo Fisher Scientific). Cell lysates were mixed with 4X Laemmli sample buffer, denatured at 95°C for 5 min, and resolved using an 8% SDS–PAGE gel (containing 0.5% 2,2,2-Trichloroethanol [TCE]), alongside protein ladders. Total protein was determined using the ChemiDoc imaging system (Bio-Rad) through UV activation. Subsequently, samples were transferred to polyvinylidene difluoride (PVDF) membranes. Membranes were blocked for 1 h at RT using a blocking buffer (2.5% BSA in Tris-buffered saline with Tween [TBST]). Membranes were incubated with primary antibody diluted in blocking buffer at 4°C overnight with agitation, and subsequently incubated with secondary antibody diluted in blocking buffer for 1 h at RT. Then, membranes were incubated with SuperSignal West Femto Maximum Sensitivity Substrate (Thermo Fisher Scientific) and visualized using the ChemiDoc imaging system (Bio-Rad). Protein band intensity was quantified using Bio-Rad Image Lab software (version 6.1).

### Capillary-based immunoassays (Simple Western)

Capillary electrophoresis using Simple Western technology (ProteinSimple) was carried out as previously described (Filippelli et al, 2022). Cells were lysed using RIPA buffer (150 nM NaCl; 10 mM Tris, pH 7.2; 0.1% SDS; 1% Triton X-100, 1% deoxycholate; 5 mM EDTA; 1X protease inhibitor, and 1X phosphatase inhibitor) on ice for 30 min, followed by centrifugation at 15,000 rcf at 4°C for 20 min. Protein lysates were mixed with Fluorescent Master Mix (EZ standard pack I, ProteinSimple). Samples, blocking reagent (Antibody Diluent 2, ProteinSimple), primary antibodies, total protein labeling reagents (Total Protein Detection Module, ProteinSimple), HRP-conjugated secondary antibodies, luminol-peroxide, RePlex reagent (RePlex Module, ProteinSimple) and wash buffer were loaded in a 12–230 kD separation module (ProteinSimple). Protein analyses were performed using the Jess Simple Western instrument (ProteinSimple). Results were analyzed using Compass for SW software (version: 6.1.0). Images from the high dynamic range exposure were used for the analysis. The area under specific protein peaks were used to determine protein quantity. Normalization between samples were performed either with the expression of house-keeping proteins (cyclophilin A, β-actin) or from the area under the entire spectrum from the total protein assay was used. Phosphorylated STAT3 was normalized to total STAT3 levels by performing the first immunoassay with phospho-STAT3 (Y705) antibody followed by the second immunoassay with STAT3 antibody within the same capillary using the RePlex Module.

### RNA isolation and digital droplet PCR

RNA was extracted using the PicoPure RNA Isolation Kit (Applied Biosystems), and cDNA was generated with the SuperScript III First-Strand Synthesis System (Invitrogen) according to the manufacturer’s protocol. Digital droplet PCR was performed using ddPCR Supermix for Probes (no dUTP) (Bio-Rad) according to the manufacturer’s protocol and analyzed with the QX100 Droplet Digital PCR System (Bio-Rad). *Ptpn1* and *Ptpn2* expression was normalized to the geometric mean of *Rps18* and *Rps20* reference genes. PrimeTime assays for *Ptpn1* (Mm.PT.58.10717153), *Ptpn2* (Mm.PT.58.14144583), *Rps18* (Mm.PT.58.12109666), and *Rps20* (Mm.PT.58.41623895.g) were purchased from Integrated DNA Technologies. Sequences for primers and probes are detailed in Table S2.

Table S2. Sequences for primers and probes used for droplet digital PCR.

### Antibodies

Primary antibodies used for IF included anti-MyHC (M4276, 1:150; MilliporeSigma); anti-PAX7 (Developmental Studies Hybridoma Bank, undiluted) (Kawakami et al, 1997); anti-MYOD (sc-377460, 1:100; Santa Cruz); anti-MYOG (NBP2-54972, 1:100; Novus); anti-STAT3 (9139, 1:300; Cell Signaling Technologies). Secondary antibodies used for IF included donkey anti-rabbit IgG (H+L) Alexa Fluor 488 (A21202, 1:1,000; Invitrogen); donkey anti-mouse IgG (H+L) Alexa Fluor 647 (A31571, 1:1,000; Invitrogen); goat anti-mouse IgG2b (y2b) Alexa Fluor 555 (A21147, 1:1,000; Invitrogen); goat anti-mouse IgG1 Alexa Fluor 647 (A21240, 1:1,000; Invitrogen); phalloidin-iFluor 488 (ab176753, 1:1,000; Abcam).

Primary antibodies used for immunoblotting analysis included anti-MyHC (M4276, 1:1,000; MilliporeSigma); anti-p-STAT3 Y705 (9145, 1:1,000; Cell Signaling Technologies); anti-STAT3 (4904, 1:1,000; Cell Signaling Technologies); anti-PTPN1 (610139, 1:10,000; BD Biosciences); anti-PTPN2 clone 3E2 (generated in house, You-Ten et al, 1997, 1:1,000). Secondary antibodies used for immunoblotting included goat anti-mouse IgG (H+L)-HRP (1706516, 1:10,000; Bio-Rad); and goat anti-rabbit IgG (H+L)-HRP (1706515, 1:10,000; Bio-Rad).

Primary antibodies used for Simple Western analysis included anti-MyHC MF 20 (1:2; Developmental Studies Hybridoma Bank) (Bader et al, 1982); anti-p-STAT3 Y705 (9145, 1:25; Cell Signaling Technologies); anti-STAT3 (4904, 1:25; Cell Signaling Technologies); anti-PTPN1 (610139, 1:25; BD Biosciences); anti-PTPN2 (1930, 1:100; R&D Systems), anti-cyclophilin A (2175, 1:50; Cell Signaling Technologies); and anti-β-actin (MAB8929, 1:500; R&D Systems). Secondary antibodies used for Simple Western included anti-rabbit HRP (042-206; ProteinSimple, ready to use); anti-mouse HRP antibody (042-205; ProteinSimple, ready to use), donkey anti-mouse IgG (H+L) Alexa Fluor 647 (A31571, 1:100; Invitrogen); and donkey anti-goat IgG (H+L) Alexa Fluor 647 (A32849, 1:100; Invitrogen).

Antibodies used for FACS included PE Rat Anti-Mouse CD31 (553373, 1:40,000; BD Biosciences); PE Rat Anti-Mouse CD45 (553081, 1:40,000; BD Biosciences); PE Rat Anti-Mouse CD11b (553311, 1:40,000; BD Biosciences); PE Rat Anti-Mouse Ly-6A/E (SCA1, 553108, 1:40,000; BD Biosciences); Rat Alexa647 anti-integrin alpha-7 (R2F2) (67-0010-05, 1:1,000; UBC AbLab); and Rat PE-Cy7 VCAM1 (CD106) (105720, 1:4,000; BioLegend).

### Statistical analysis

Statistical tests were performed using GraphPad Prism software (v10.0.2). Statistical tests are as indicated. One-way ANOVA was performed for all within-group differentiation assays. Two-way ANOVAs were used for between group comparisons. Post-hoc testing was performed using Fisher’s LSD because of low *K*-values. Pearson’s correlation was used for Fig S2A and C. All other statistics were calculated using two-sided unpaired *t* tests.

## Supplementary Material

Reviewer comments

## Data Availability

All data underlying the research presented in the manuscript are available in the published article and its online supplemental material.
