# Supplementary material for: PTPN1/2 inhibition promotes muscle stem cell differentiation in Duchenne muscular dystrophy
Source: Life Sci Alliance. 2024 Oct 30;8(1):e202402831. doi: 10.26508/lsa.202402831 (PMC11527974; doi:10.26508/lsa.202402831)
Supplement: Supplementary file 1 [file LSA-2024-02831_SdataF1_F2_F4_F5_F6.pdf]

Source Data

Figure 1B

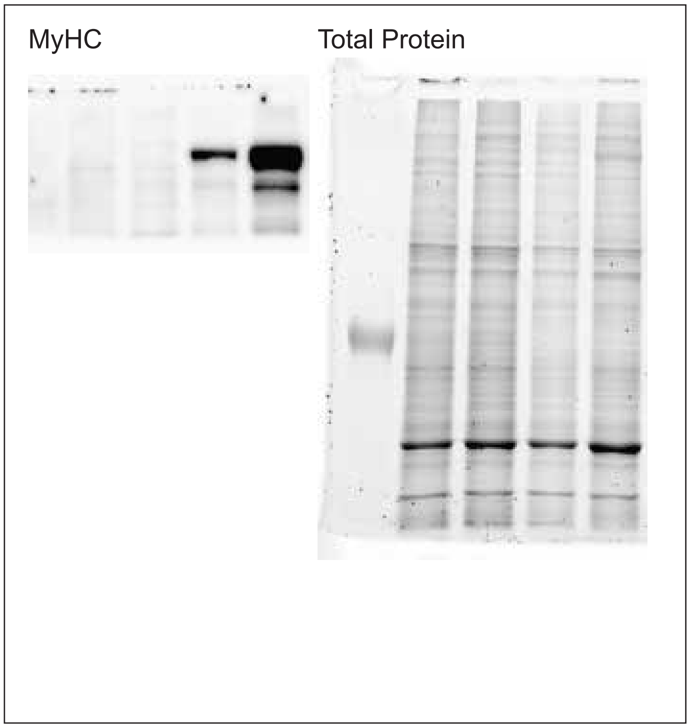

Figure 1B and Figure S1A

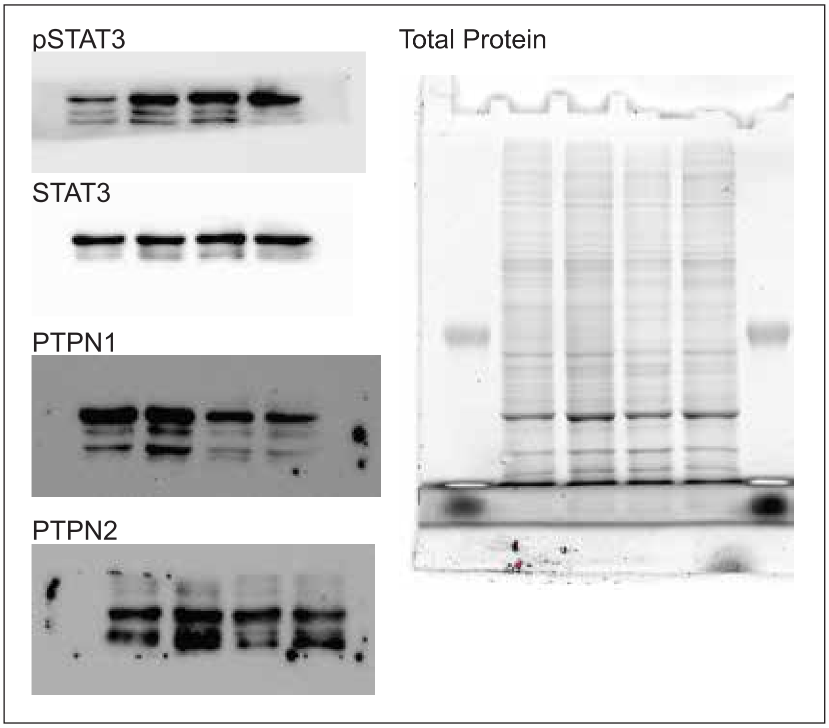

Figure 1E

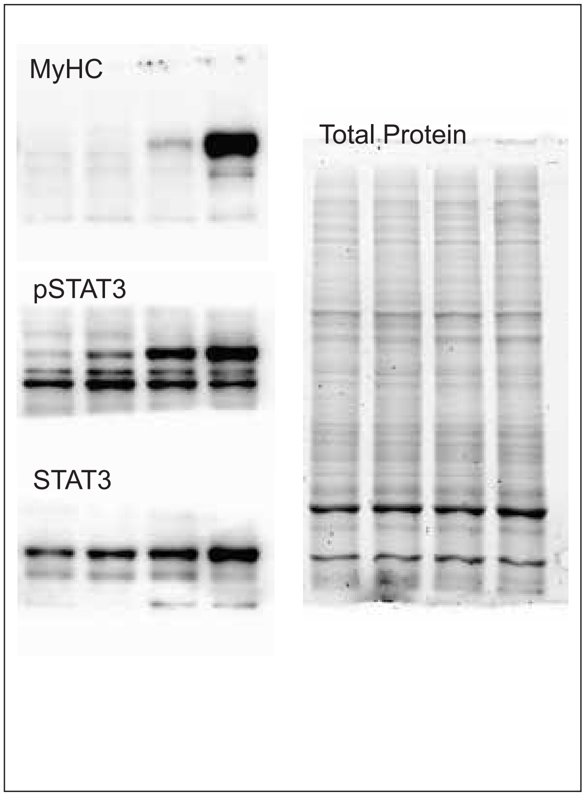

Figure 1E

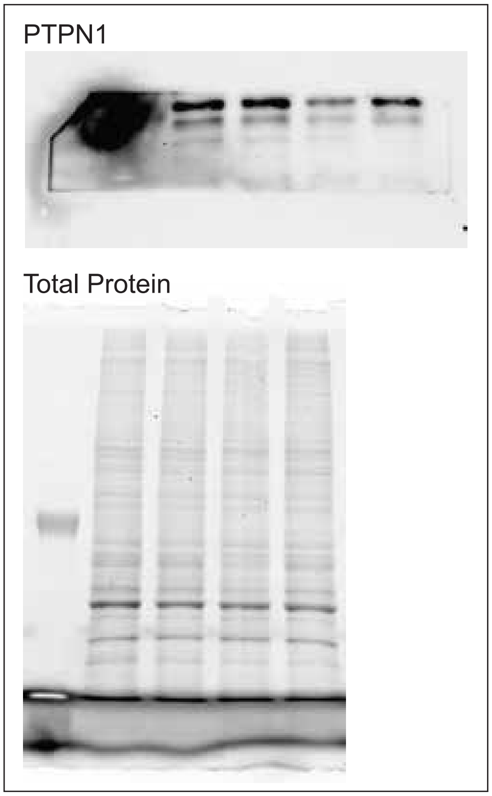

Figure S1B

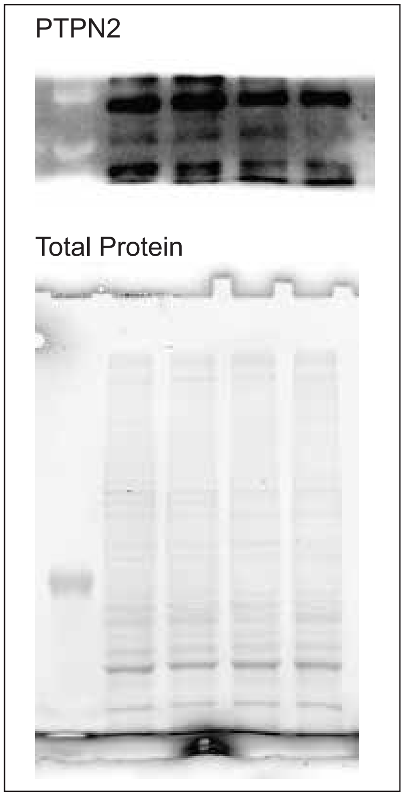

Figure 2C

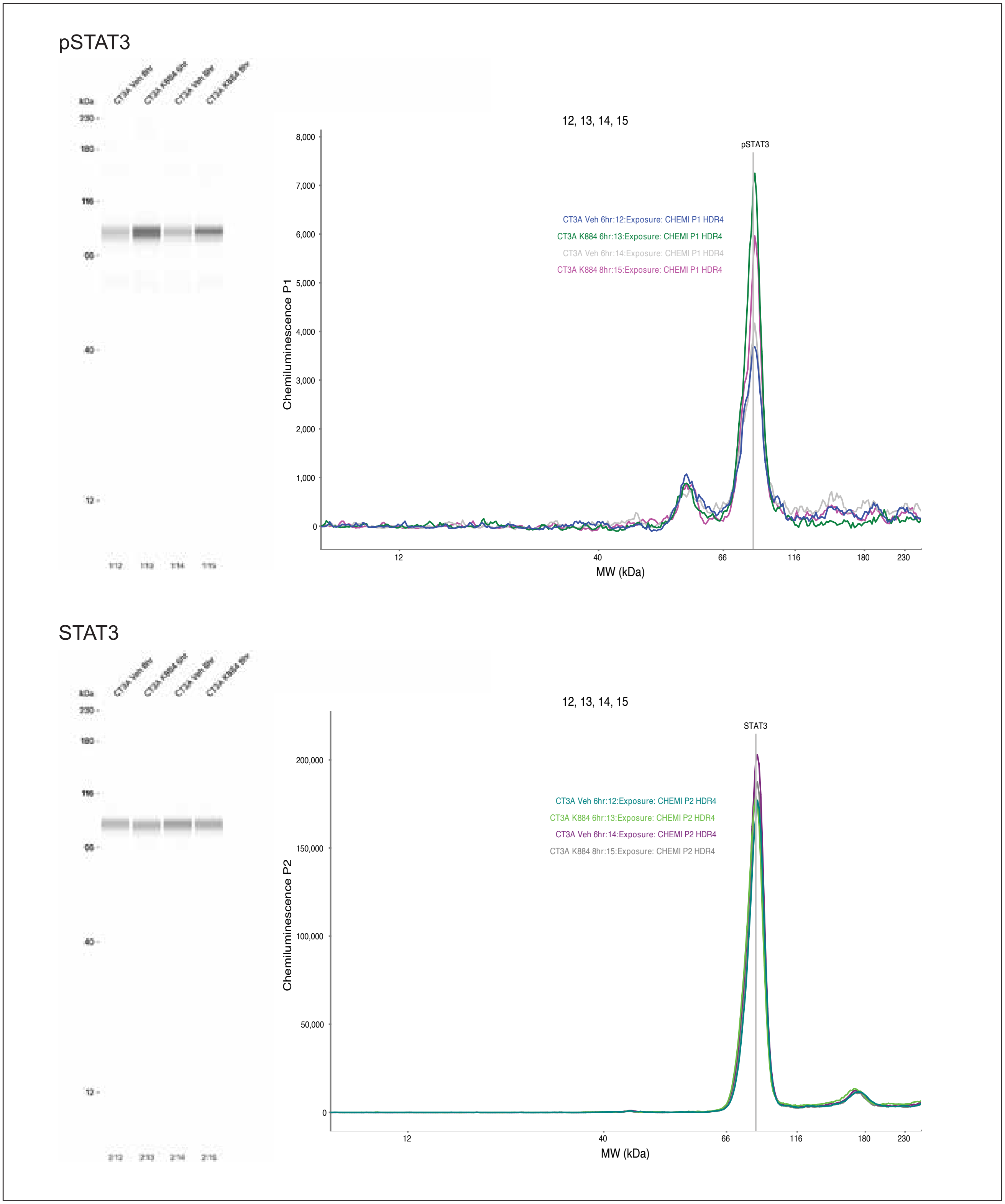
pSTAT3
12, 13, 14, 15
pSTAT3
CT3A Veh 6hr:12:Exposure: CHEMI P1 HDR4
CT3A K884 6hr:13:Exposure: CHEMI P1 HDR4
CT3A Veh 6hr:14:Exposure: CHEMI P1 HDR4
CT3A K884 8hr:15:Exposure: CHEMI P1 HDR4
Chemiluminescence P1
8,000
7,000
6,000
5,000
4,000
3,000
2,000
1,000
0
12
40
66
116
180
230
MW (kDa)
STAT3
12, 13, 14, 15
STAT3
CT3A Veh 6hr:12:Exposure: CHEMI P2 HDR4
CT3A K884 6hr:13:Exposure: CHEMI P2 HDR4
CT3A Veh 6hr:14:Exposure: CHEMI P2 HDR4
CT3A K884 8hr:15:Exposure: CHEMI P2 HDR4
Chemiluminescence P2
200,000
150,000
100,000
50,000
0
12
40
66
116
180
230
MW (kDa)

Figure 2D

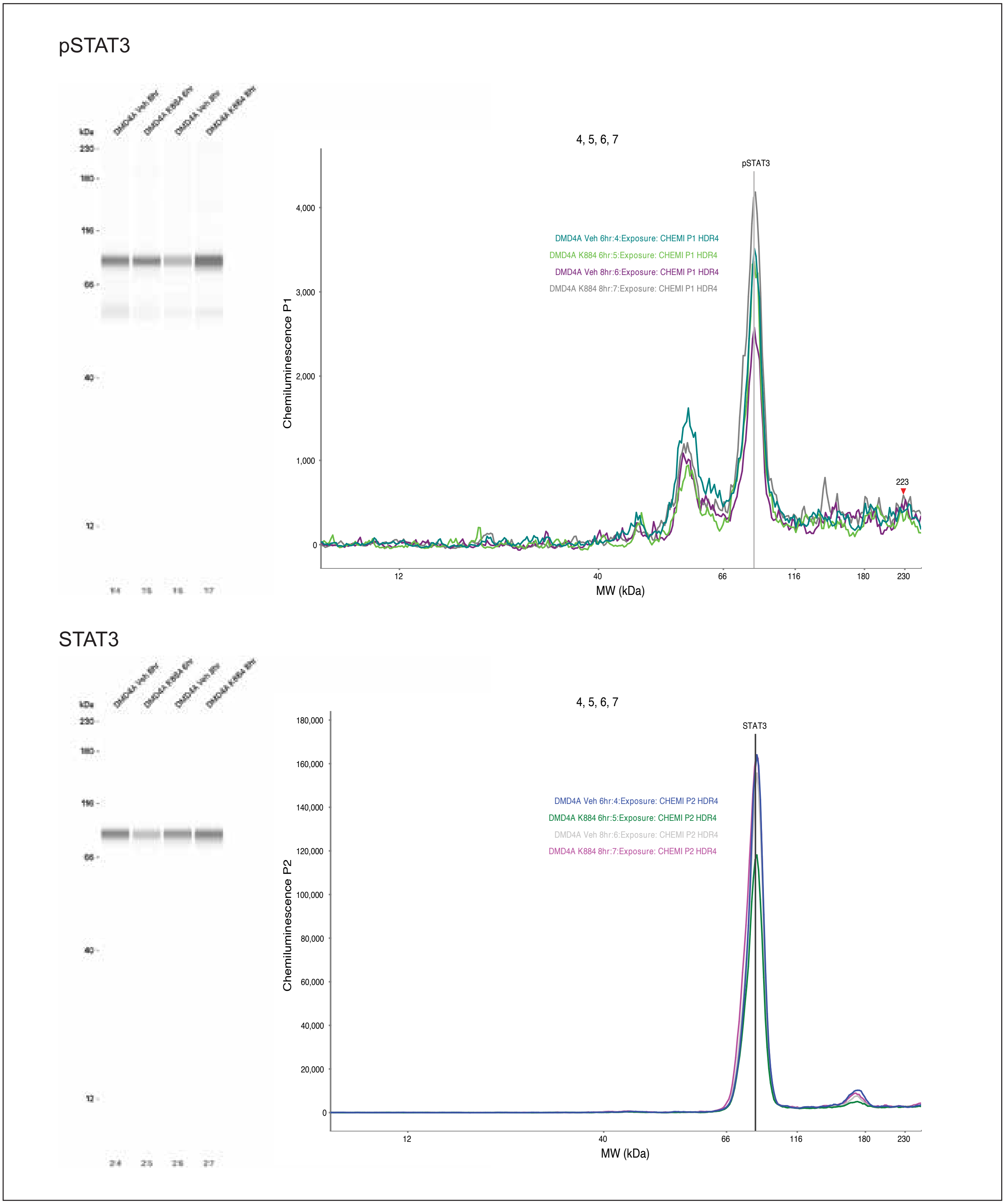
pSTAT3
4, 5, 6, 7
pSTAT3
DMD4A Veh 6hr:4:Exposure: CHEMI P1 HDR4
DMD4A K884 6hr:5:Exposure: CHEMI P1 HDR4
DMD4A Veh 8hr:6:Exposure: CHEMI P1 HDR4
DMD4A K884 8hr:7:Exposure: CHEMI P1 HDR4
Chemiluminescence P1
4,000
3,000
2,000
1,000
0
223
12
40
66
116
180
230
MW (kDa)
STAT3
4, 5, 6, 7
STAT3
DMD4A Veh 6hr:4:Exposure: CHEMI P2 HDR4
DMD4A K884 6hr:5:Exposure: CHEMI P2 HDR4
DMD4A Veh 8hr:6:Exposure: CHEMI P2 HDR4
DMD4A K884 8hr:7:Exposure: CHEMI P2 HDR4
Chemiluminescence P2
180,000
160,000
140,000
120,000
100,000
80,000
60,000
40,000
20,000
0
12
40
66
116
180
230
MW (kDa)

Figure 4A

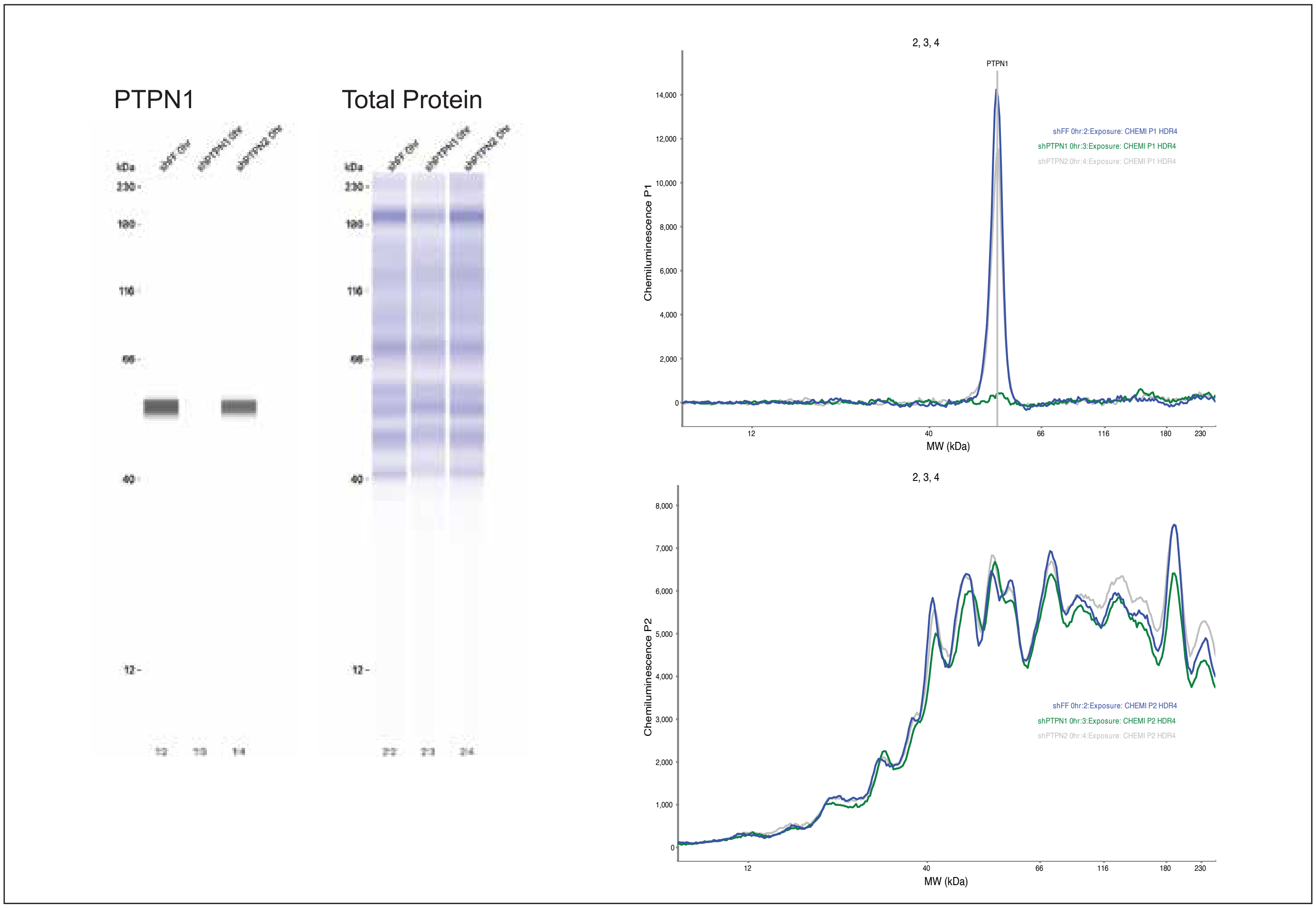

Figure 4B

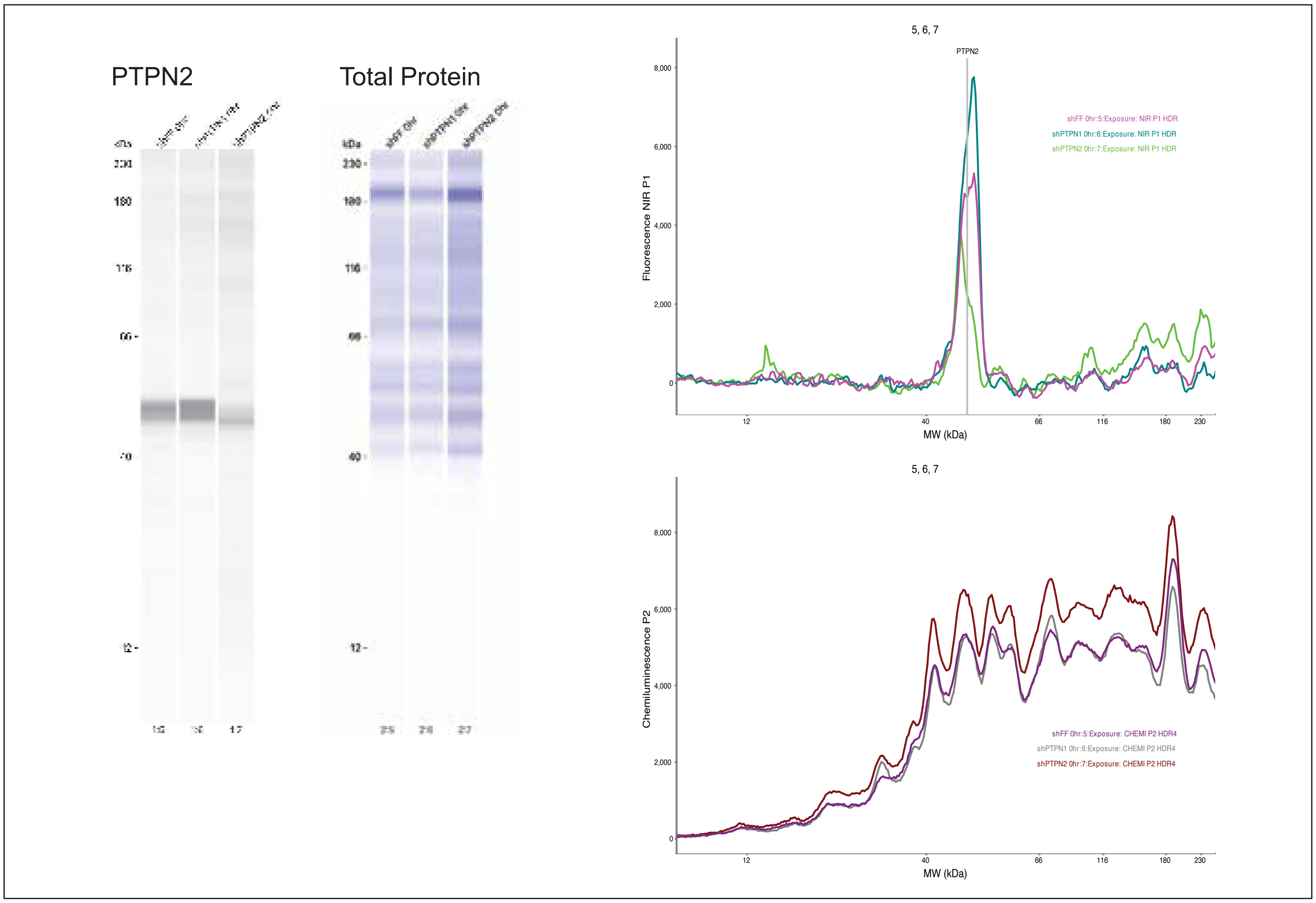

Figure 4C

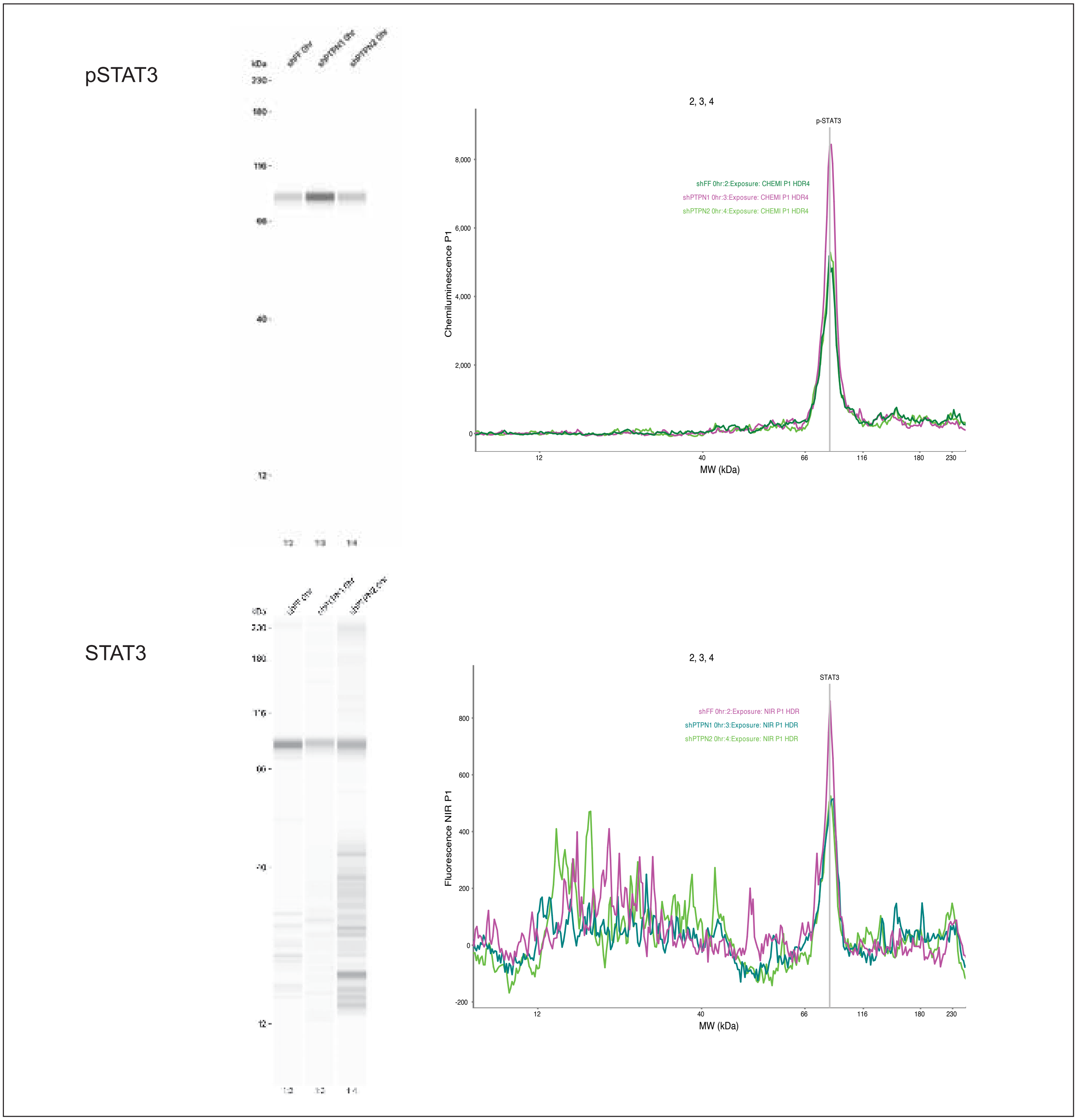
pSTAT3
STAT3
2, 3, 4
p-STAT3
shFF 0hr:2:Exposure: CHEMI P1 HDR4
shPTPN1 0hr:3:Exposure: CHEMI P1 HDR4
shPTPN2 0hr:4:Exposure: CHEMI P1 HDR4
Chemiluminescence P1
MW (kDa)
2, 3, 4
STAT3
shFF 0hr:2:Exposure: NIR P1 HDR
shPTPN1 0hr:3:Exposure: NIR P1 HDR
shPTPN2 0hr:4:Exposure: NIR P1 HDR
Fluorescence NIR P1
MW (kDa)

Figure 4D

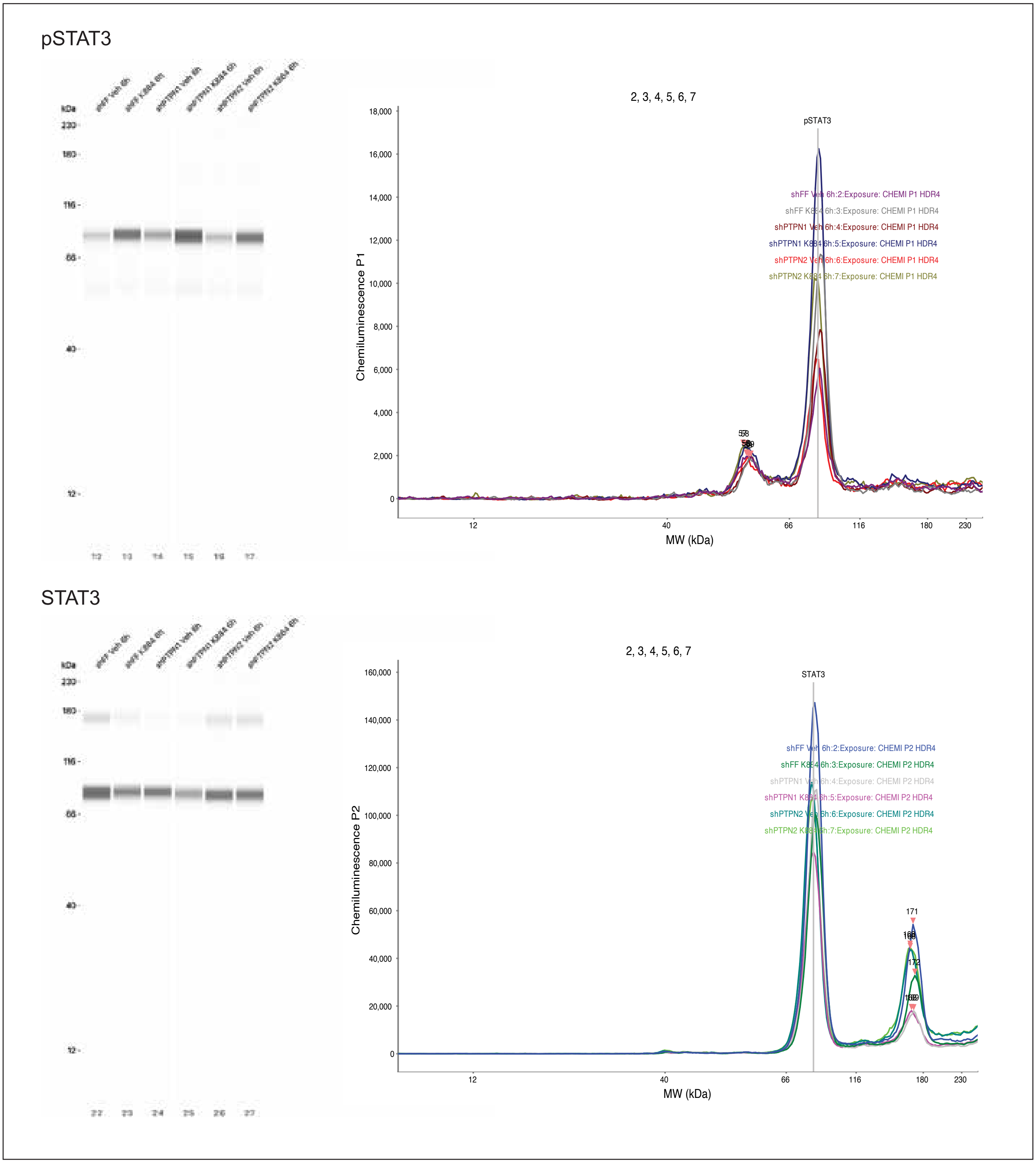

pSTAT3
2, 3, 4, 5, 6, 7
Chemiluminescence P1
MW (kDa)
shFF Veh 6h:2:Exposure: CHEMI P1 HDR4
shFF K884 6h:3:Exposure: CHEMI P1 HDR4
shPTPN1 Veh 6h:4:Exposure: CHEMI P1 HDR4
shPTPN1 K884 6h:5:Exposure: CHEMI P1 HDR4
shPTPN2 Veh 6h:6:Exposure: CHEMI P1 HDR4
shPTPN2 K884 6h:7:Exposure: CHEMI P1 HDR4
STAT3
2, 3, 4, 5, 6, 7
Chemiluminescence P2
MW (kDa)
shFF Veh 6h:2:Exposure: CHEMI P2 HDR4
shFF K884 6h:3:Exposure: CHEMI P2 HDR4
shPTPN1 Veh 6h:4:Exposure: CHEMI P2 HDR4
shPTPN1 K884 6h:5:Exposure: CHEMI P2 HDR4
shPTPN2 Veh 6h:6:Exposure: CHEMI P2 HDR4
shPTPN2 K884 6h:7:Exposure: CHEMI P2 HDR4

Figure 4E

MyHC

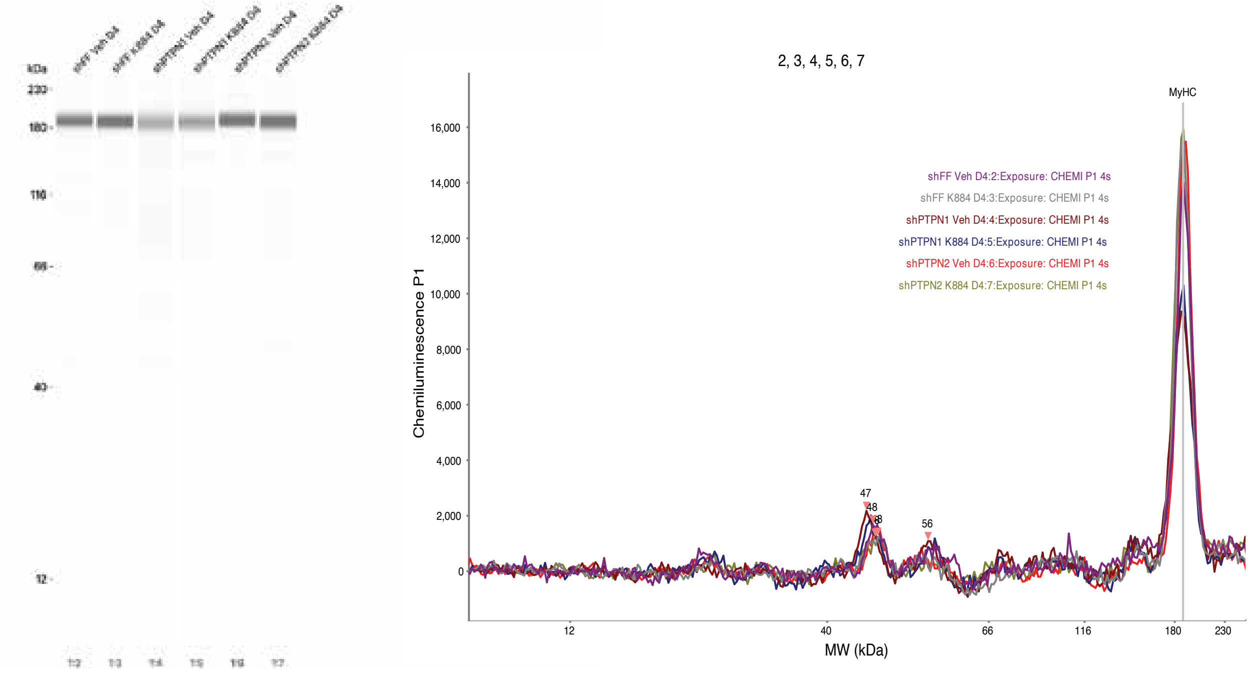

Total Protein

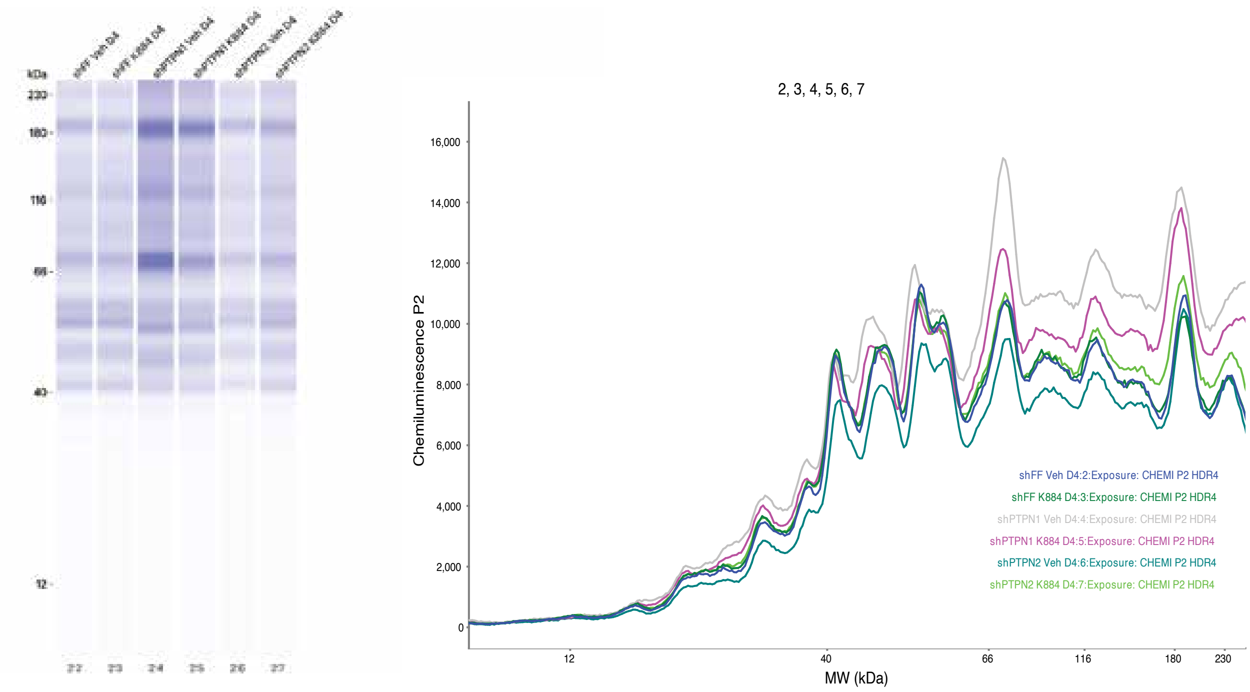

Figure 5C

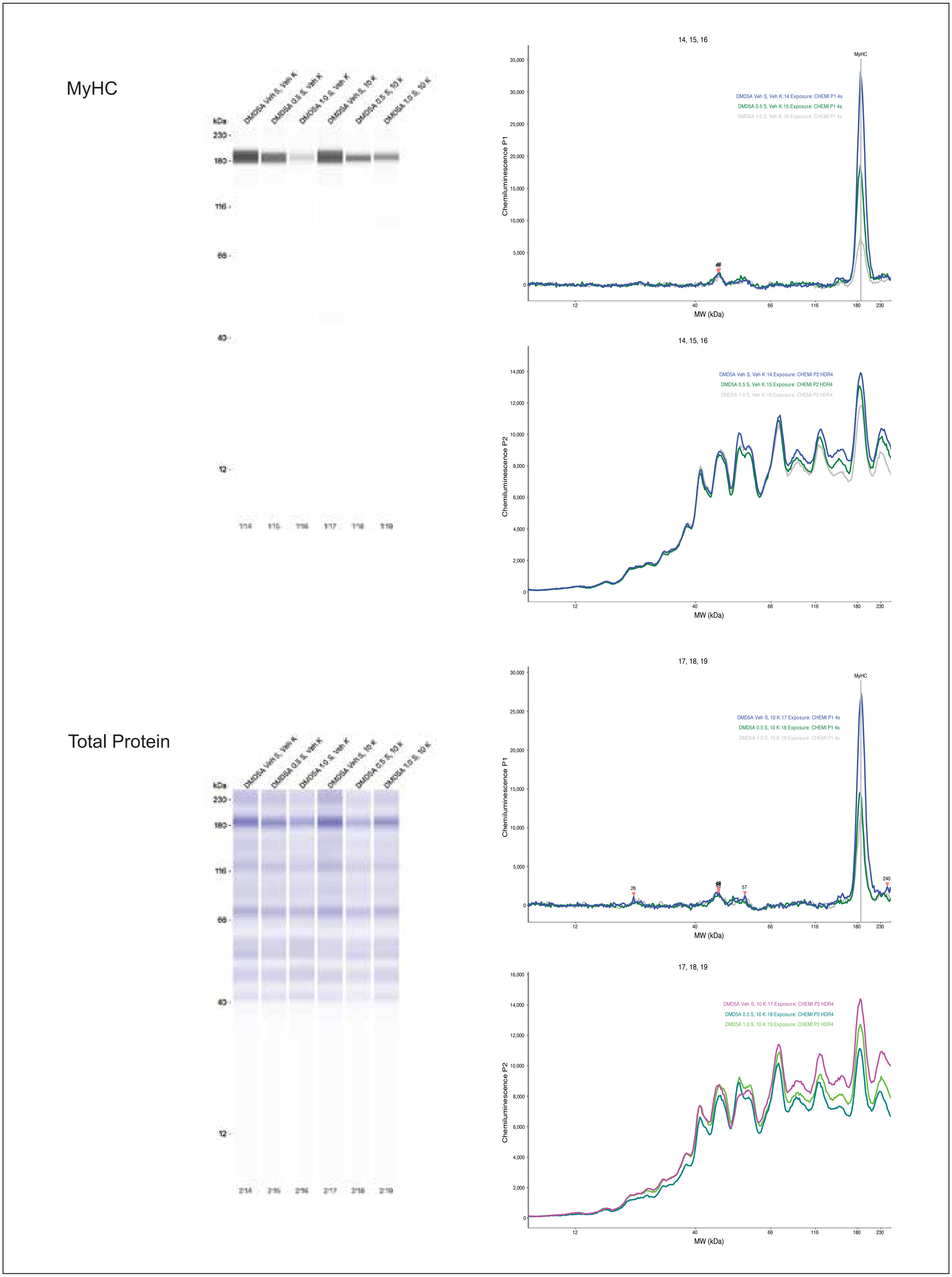
MyHC
Total Protein
14, 15, 16
MyHC
DMD5A Veh S, Veh K:14:Exposure: CHEMI P1 4s
DMD5A 0.5 S, Veh K:15:Exposure: CHEMI P1 4s
DMD5A 1.0 S, Veh K:16:Exposure: CHEMI P1 4s
Chemiluminescence P1
MW (kDa)
14, 15, 16
DMD5A Veh S, Veh K:14:Exposure: CHEMI P2 HDR4
DMD5A 0.5 S, Veh K:15:Exposure: CHEMI P2 HDR4
DMD5A 1.0 S, Veh K:16:Exposure: CHEMI P2 HDR4
Chemiluminescence P2
MW (kDa)
17, 18, 19
MyHC
DMD5A Veh S, 10 K:17:Exposure: CHEMI P1 4s
DMD5A 0.5 S, 10 K:18:Exposure: CHEMI P1 4s
DMD5A 1.0 S, 10 K:19:Exposure: CHEMI P1 4s
Chemiluminescence P1
MW (kDa)
17, 18, 19
DMD5A Veh S, 10 K:17:Exposure: CHEMI P2 HDR4
DMD5A 0.5 S, 10 K:18:Exposure: CHEMI P2 HDR4
DMD5A 1.0 S, 10 K:19:Exposure: CHEMI P2 HDR4
Chemiluminescence P2
MW (kDa)

Figure 6B

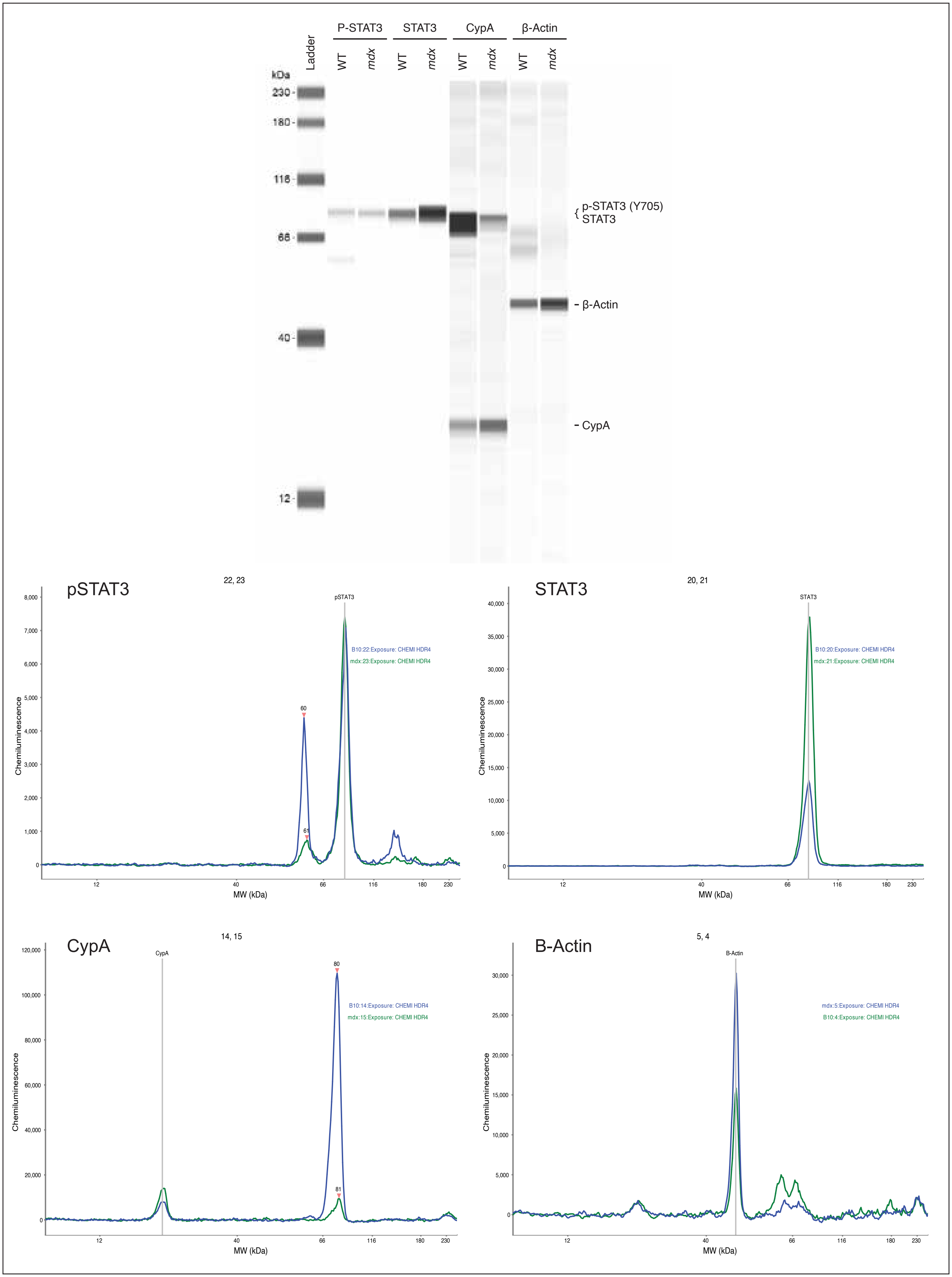

P-STAT3
STAT3
CypA
β-Actin
Ladder
WT
mdx
kDa
230
180
116
66
40
12
p-STAT3 (Y705)
STAT3
β-Actin
CypA
pSTAT3
22, 23
B10:22:Exposure: CHEMI HDR4
mdx:23:Exposure: CHEMI HDR4
Chemiluminescence
MW (kDa)
20, 21
B10:20:Exposure: CHEMI HDR4
mdx:21:Exposure: CHEMI HDR4
14, 15
B10:14:Exposure: CHEMI HDR4
mdx:15:Exposure: CHEMI HDR4
B-Actin
5, 4
mdx:5:Exposure: CHEMI HDR4
B10:4:Exposure: CHEMI HDR4
