# Supplementary material for: PTPN1/2 inhibition promotes muscle stem cell differentiation in Duchenne muscular dystrophy
Source: Life Sci Alliance. 2024 Oct 30;8(1):e202402831. doi: 10.26508/lsa.202402831 (PMC11527974; doi:10.26508/lsa.202402831)
Supplement: Supplementary file 2 [file LSA-2024-02831_TableS1.docx]

**Tables**

| **shRNA** | **Sequences** |
| --- | --- |
| shPTPN1 | TGCTGTTGACAGTGAGCGCCCCTGTTATCTGCTAGATCTATAGTGA AGCCACAGATGTATAGATCTAGCAGATAACAGGGATGCCTACTGC CTCGGA |
| shPTPN2 | TGCTGTTGACAGTGAGCGCACAGTACATCTACTACAATTATAGTGA AGCCACAGATGTATAATTGTAGTAGATGTACTGTATGCCTACTGCC TCGGA |
| shFF | TGCTGTTGACAGTGAGCGACCGCCTGAAGTCTCTGATTAATAGTGA AGCCACAGATGTATTAATCAGAGACTTCAGGCGGGTGCCTACTGCC TCGGA |

**Supplemental Table 1**. Sequences for individual shRNAs.
