# Supplementary material for: PTPN1/2 inhibition promotes muscle stem cell differentiation in Duchenne muscular dystrophy
Source: Life Sci Alliance. 2024 Oct 30;8(1):e202402831. doi: 10.26508/lsa.202402831 (PMC11527974; doi:10.26508/lsa.202402831)
Supplement: Supplementary file 3 [file LSA-2024-02831_TableS2.docx]

| *Ptpn1* | Primer 1 | ACAGTACGACAGTTGGAGTTG |
| --- | --- | --- |
|  | Primer 2 | CTCCAAAGTCAGGCCATGT |
|  | Probe | /56-FAM/CTCTCGAGT/ZEN/CTCCTTGGTAGTCAGGTT/3IABkFQ/ |
| *Ptpn2* | Primer 1 | TGTCATGCTAAACCGAACTGT |
|  | Primer 2 | CACACTGAATCCCGTTTCCTTA |
|  | Probe | /5HEX/ACCATTTCT/ZEN/CTGTCATCCGTTGGCC/3IABkFQ/ |
| *Rps18* | Primer 1 | ACACCACATGAGCATATCTCC |
|  | Primer 2 | CCTGAGAAGTTCCAGCACAT |
|  | Probe | /5HEX/AGCCTTCGC/ZEN/CATCACTGCCATTA/3IABkFQ/ |
| *Rps20* | Primer 1 | AGTCCTTCTGAGATTGTTAAGCAG |
|  | Primer 2 | CGATTTATTCAGTTGTCTTAGGCATC |
|  | Probe | /5FAM/CGGGAGTTG/ZEN/AGGTTGAAGTCACCA/3IABkFQ/ |

**Supplemental Table 2**. Sequences for primers and probes used for droplet digital PCR.
